# A cathelicidin antimicrobial peptide from *Hydrophis cyanocinctus* inhibits Zika virus infection by downregulating expression of a viral entry factor

**DOI:** 10.1016/j.jbc.2022.102471

**Published:** 2022-09-08

**Authors:** Jing Wang, Bingyan Jiang, Kezhen Wang, Jianfeng Dai, Chunsheng Dong, Yipeng Wang, Peng Zhang, Min Li, Wei Xu, Lin Wei

**Affiliations:** 1 Jiangsu Provincial Key Laboratory of Infection and Immunity, Institutes of Biology and Medical Sciences, Soochow University, Suzhou, Jiangsu, China; 2 Department of Biopharmaceutical Sciences, College of Pharmaceutical Sciences, Soochow University, Suzhou, Jiangsu, China; 3 The Second Affiliated Hospital of Soochow University, Suzhou, China

**Keywords:** antimicrobial peptide, cathelicidin, Zika virus, AXL, AC, adenylate cyclase, AMP, antimicrobial peptide, COX-2, cyclo-oxygenase-2, DMEM, dulbecco’s modified eagle’s medium, FBS, fetal bovine serum, FSK, forskolin, Hc-CATH, cathelicidin antimicrobial peptide identified from the sea snake *Hydrophis cyanocinctus*, IF, immunofluorescence, IFN, interferon, MOI, multiplicity of infection, PFU, plaque-forming unit, PGE_2_, prostaglandin E_2_, IRF3, interferon regulatory factor 3, PS, phosphatidylserine, qPCR, quantitative PCR, SeV, sendai virus, TIM, T-cell immunoglobulin and mucin domain, TAM, TYRO3, AXL, and MERTK family, WB, western blot, ZIKV, Zika virus

## Abstract

Zika virus (ZIKV) is a re-emerging flavivirus that causes conditions such as microcephaly and testis damage. The spread of ZIKV has become a major public health concern. Recent studies indicated that antimicrobial peptides are an ideal source for screening antiviral candidates with broad-spectrum antiviral activities, including against ZIKV. We herein found that Hc-CATH, a cathelicidin antimicrobial peptide identified from the sea snake *Hydrophis cyanocinctus* in our previous work, conferred protection against ZIKV infection in host cells and showed preventative efficacy and therapeutic efficacy in C57BL/6J mice, *Ifnar1*^−/−^ mice, and pregnant mice. Intriguingly, we revealed that Hc-CATH decreased the susceptibility of host cells to ZIKV by downregulating expression of AXL, a TAM (TYRO3, AXL and MERTK) family kinase receptor that mediates ZIKV infection, and subsequently reversed the negative regulation of AXL on host’s type I interferon response. Furthermore, we showed that the cyclo-oxygenase-2/prostaglandin E2/adenylyl cyclase/protein kinase A pathway was involved in Hc-CATH-mediated AXL downregulation, and Hc-CATH in addition directly inactivated ZIKV particles by disrupting viral membrane. Finally, while we found Hc-CATH did not act on the late stage of ZIKV infection, structure–function relationship studies revealed that α-helix and phenylalanine residues are key structural requirements for its protective efficacy against initial ZIKV infection. In summary, we demonstrate that Hc-CATH provides prophylactic and therapeutic efficacy against ZIKV infection *via* downregulation of AXL, as well as inactivating the virion. Our findings reveal a novel mechanism of cathelicidin against viral infection and highlight the potential of Hc-CATH to prevent and treat ZIKV infection.

Zika virus (ZIKV) is a single-stranded, enveloped, and positive-sense RNA virus belonging to the Flaviviridae family (1). This group of approximately 70 viruses include members such as dengue virus, yellow fever virus, West Nile virus, Japanese encephalitis virus, and tick-borne encephalitis virus (2, 3). In all flaviviruses, the RNA genome encodes a polyprotein that is processed into three structural proteins (core C, membrane precursor prM, and envelope E) and seven nonstructural proteins (NS1, NS2A, NS2B, NS3, NS4A, NS4B, and NS5) (1, 4). The structural proteins form the virus particle and the nonstructural proteins assist in replication and packaging of the genome. *Aedes* mosquitoes are the main vectors for the transmission of ZIKV (5). Besides, ZIKV can also be transmitted through sexual, vertical, and blood transmission (6, 7). Initially, ZIKV did not arouse widespread concern in society, and there were few related cases of ZIKV (8). Recent epidemiological studies have shown that ZIKV infection can cause a variety of serious diseases, which can lead to microcephaly, congenital malformations, brain calcification, fetal death, multiple organ failure, thrombocytopenia, thrombocytopenic purpura, testis damage, and male infertility (9, 10). In addition, ZIKV infection can also cause blindness and a variety of eye abnormalities, including retinal spots, lens subluxation, and optic neuritis (2, 11). Therefore, it is urgent to develop safe and effective drugs for the prevention and therapy of ZIKV infection.

Many molecules expressed on the cell surface are involved in viral infection, including the DC cell surface c-type lectin receptor superfamily (DC-SING), DC-SING-related proteins (L-SING) (12, 13), and phosphatidylserine (PS) receptors (11, 14). PS receptors play a critical role in flavivirus infection, including members of the TIM (T-cell immunoglobulin and mucin domain) family and TAM (TYRO3, AXL and MERTK) family (15, 16). TAM receptors belong to the tyrosine receptor family, a transmembrane protein that transmits signals from the extracellular environment to the cytoplasm and nucleus. TAM receptors regulate various cellular functions, including clearance of apoptotic cells by macrophages, platelet aggregation, and differentiation of natural killer cells (17). AXL belongs to the TAM family and can be expressed in brain and neural progenitor cells. It has been reported that AXL plays an important role in ZIKV infection (18, 19, 20, 21). On the one hand, AXL can act as a cofactor for the entry of ZIKV to host cells (15, 22, 23). On the other hand, AXL can regulate the innate immune response of host cells by negatively regulating interferon (IFN) signaling and the expression of inflammatory factors, thus promoting ZIKV infection (22, 24, 25). These molecules involved in viral infection are potential targets for the prevention and therapy of ZIKV infection.

Antimicrobial peptides (AMPs), also called host defense peptides, are a group of relatively small cationic peptides. They are an important part of the innate immune system and play an important role in fighting against bacterial infection (26). Recently, more and more studies have shown that AMPs can also exert antiviral effects by inhibiting virus adsorption, disrupting virus envelope, and regulating host antiviral immune response (27, 28, 29). AMPs have become an important source for screening antiviral peptides with broad antiviral spectrum against herpes simplex virus (30, 31), human immunodeficiency virus (32), hepatitis C virus (33), and influenza A virus (34). Therefore, AMPs can be used as new candidates for the development of antiviral drugs. In mammals, the two major AMP families are cathelicidins and defensins, among which cathelicidins have direct antimicrobial activity against a variety of microorganisms and actively participate in host’s immune regulation (35). Cathelicidins are also found in the venom of various snakes. Previous studies have shown that cathelicidins derived from snakes have strong antibacterial activity with broad spectrum, including clinically isolated drug-resistant bacterial strains (36, 37). Besides, snake-derived cathelicidins have extremely low cytotoxicity and hemolytic activity, showing a high clinical application prospect. In our previous study, we identified a novel cathelicidin peptide from the venom of the sea snake, *Hydrophis cyanocinctus*, which is designated as Hc-CATH (26). It showed a broad antibacterial spectrum as well as potent anti-inflammatory activity with high stability and low cytotoxicity (26). However, few attention was paid to its antiviral activity. In this study, we tried to evaluate the effects of Hc-CATH on ZIKV infection, including its effect on ZIKV-caused cytopathic effect, ZIKV replication *in vitro*, and ZIKV replication *in vivo*. The mechanism of action against ZIKV infection was also investigated.

## Results

### Hc-CATH inhibits ZIKV infection *in vitro*

**Figure 1:**
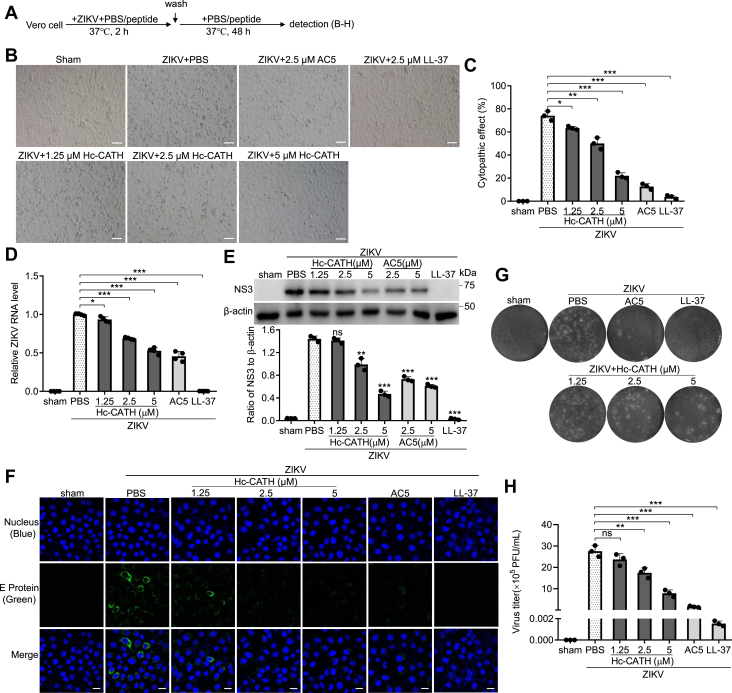
**Hc-CATH inhibits ZIKV infection *in vitro***. *A*, schematic diagram of *B*–*H*. *B*–*H*, protective effect of Hc-CATH on ZIKV infection *in vitro*. Vero cells were seeded in 24-well plates (5 × 10^4^ cells/well). Cells were infected with ZIKV (MOI = 1), and Hc-CATH (1.25, 2.5, and 5 μM), AC5 (2.5 and 5 μM), LL-37 (2.5 μM), or same volume of PBS (peptide solvent) was added and incubated at 37 °C for 2 h. Then the cells were washed with PBS and transferred with fresh DMEM containing 2% FBS and the same concentration of peptides. After culture at 37 °C for 48 h, image of ZIKV-caused cytopathic effect was taken (*B*, the scale bar represents 200 μm), the percentage of ZIKV-caused cytopathic effect was quantified by CCK-8 (*C*), intracellular ZIKV RNA (*D*), NS3 protein (*E*, *upper panel*: immunoblots, *lower panel*: ratios analyzed by ImageJ), E protein (*F*, the scale bar represents 50 μm), and extracellular ZIKV titer (*G* and *H*) was tested by qPCR, Western blot, immunofluorescence staining, and plaque-forming assay, respectively. The raw data files used in the creation of *G* are presented in Fig. S3. ∗*p* < 0.05, ∗∗*p* < 0.01, ∗∗∗*p* < 0.001, ns, not significant. CCK-8, Cell Counting Kit-8; DMEM, Dulbecco’s modified Eagle’s medium; FBS, fetal bovine serum; Hc-CATH, a cathelicidin antimicrobial peptide identified from the sea snake *Hydrophis cyanocinctus*; MOI, multiplicity of infection; qPCR, quantitative PCR; ZIKV, Zika virus.

In order to explore whether Hc-CATH has antiviral activity against ZIKV, Vero cells were infected with ZIKV in the presence of Hc-CATH or PBS (solvent of peptide) as indicated in Fig. 1*A*, and the effect of Hc-CATH on ZIKV-caused cytopathic effect and ZIKV replication were detected. As shown in Fig. 1, *B* and *C*, ZIKV infection obviously caused cytopathic effect in Vero cells, and Hc-CATH markedly reversed the cytopathic effect caused by ZIKV infection in a dose-dependent manner. Hc-CATH also reduced the intracellular ZIKV RNA (Fig. 1*D*), NS3 protein (Fig. 1*E*), E protein (Fig. 1*F*), and extracellular ZIKV particles (Fig. 1, *G* and *H*) in a dose-dependent manner. At concentration of 5 μM, Hc-CATH reduced about 70.3% cytopathic effect, 52.7% ZIKV RNA, 67.4% NS3 protein, and 71.6% ZIKV particles. Besides, Hc-CATH did not have cytotoxicity against mammalian cells at concentrations of 1.25, 2.5, and 5 μM (Fig. S1). The results indicate that Hc-CATH shows inhibitory effect against ZIKV infection in Vero cells.

### Hc-CATH decreases the susceptibility of host cells to ZIKV

**Figure 2:**
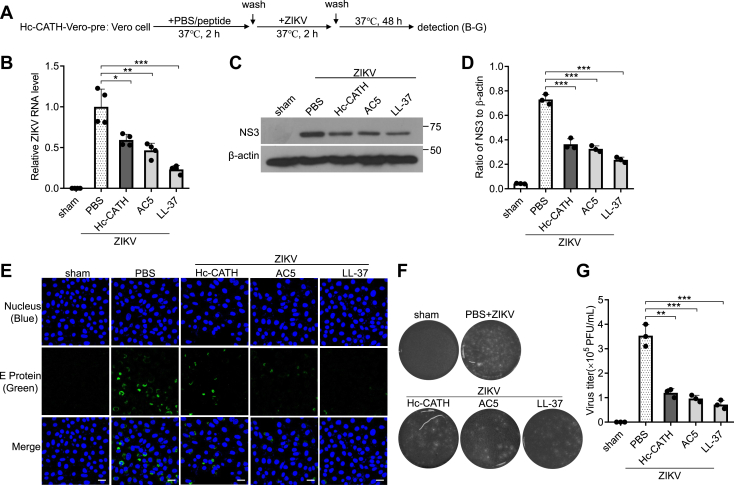
**Hc-CATH decreases the susceptibility of Vero cells to ZIKV.***A*, schematic diagram of *B*–*G* (Hc-CATH-Vero-pre). *B*–*G*, pretreatment with Hc-CATH before ZIKV infection decreases the susceptibility of Vero cells to ZIKV. Vero cells were seeded in 24-well plates (5 × 10^4^ cells/well) and cultured in DMEM containing 2% FBS. Hc-CATH (2.5 μM), AC5 (2.5 μM), LL-37 (2.5 μM), or same volume of PBS (peptide solvent) was added to cells and incubated at 37 °C for 2 h. Vero cells were washed three times with PBS, and ZIKV (MOI = 1) was added to cells. After incubation at 37 °C for 2 h, Vero cells were washed with PBS and transferred with fresh DMEM containing 2% FBS. After culture for 48 h, intracellular ZIKV RNA (*B*), NS3 protein (*C* and *D*, *C*: immunoblots, *D*: ratio analyzed by ImageJ), E protein (*E*, the scale bar represents 50 μm), and extracellular ZIKV titer (*F* and *G*) was tested by qPCR, Western blot, immunofluorescence staining, and plaque-forming assay, respectively. The raw data files used in the creation of *F* are presented in Fig. S4. ∗*p* < 0.05, ∗∗*p* < 0.01, and ∗∗∗*p* < 0.001. DMEM, Dulbecco’s modified Eagle’s medium; Hc-CATH, a cathelicidin antimicrobial peptide identified from the sea snake *Hydrophis cyanocinctus*; FBS, fetal bovine serum; MOI, multiplicity of infection; qPCR, quantitative PCR; ZIKV, Zika virus.

To understand the anti-ZIKV mechanism of Hc-CATH, we first investigated whether Hc-CATH decreases the susceptibility of host cells. Vero cells were incubated with Hc-CATH or PBS before ZIKV infection as indicated in Fig. 2*A* (Hc-CATH-cell-pre). As shown in Fig. 2, *B*–*G*, the incubation of Vero cells with Hc-CATH for 2 h significantly reduced the intracellular ZIKV RNA (Fig. 2*B*), NS3 protein (Fig. 2, *C* and *D*), E protein (Fig. 2*E*), and extracellular ZIKV particles (Fig. 2, *F* and *G*) relative to PBS incubation. The incubation of Vero cells with Hc-CATH reduced about 46.1% ZIKV RNA (Figs. 2*B*), 50.1% ZIKV NS3 protein (Fig. 2, *C* and *D*), and 62.2% ZIKV particles (Fig. 2, *F* and *G*). The results indicate that incubation of Vero cells with Hc-CATH decreases the susceptibility of host cells to ZIKV.

### Hc-CATH downregulates AXL in host cells

**Figure 3:**
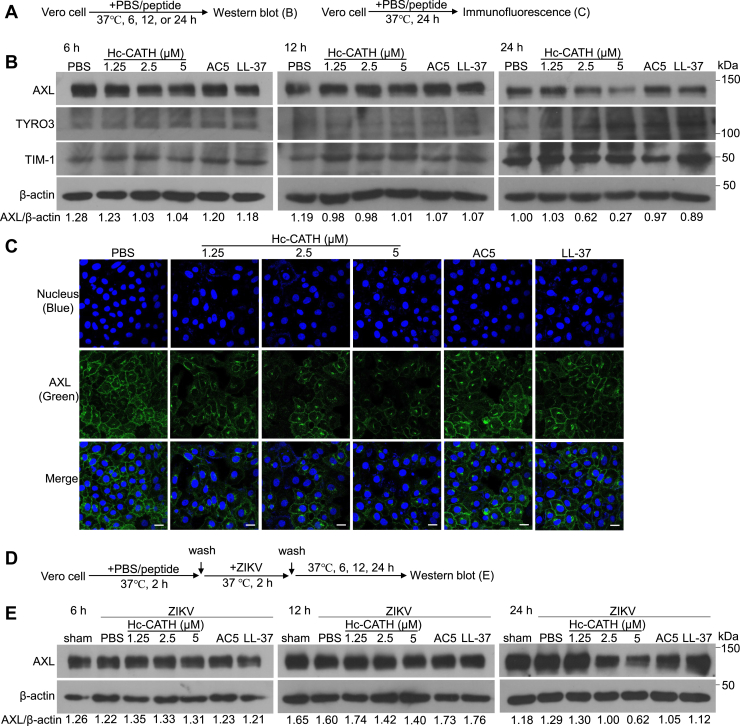
**Hc-CATH downregulates AXL in Vero cell.***A*, schematic diagram of *B* and *C*. *B*, Vero cells were seeded in 24-well plates (5 × 10^4^ cells/well) and cultured in DMEM containing 2% FBS. Hc-CATH (1.25, 2.5, and 5 μM), AC5 (2.5 μM), LL-37 (2.5 μM), or same volume of PBS (peptide solvent) was added to cells. After culture at 37 °C for 6, 12, and 24 h, the levels of ZIKV entry factors, including AXL, TYRO3, and TIM-1, were tested by Western blot, respectively. *C*, Vero cells were seeded in 8-well cover slip chambers (5 × 10^4^ cells/well) and cultured in DMEM containing 2% FBS. Hc-CATH (1.25, 2.5, and 5 μM), AC5 (2.5 μM), LL-37 (2.5 μM), or same volume of PBS (peptide solvent) was added to cells. After culture at 37 °C for 24 h, the level of AXL was tested by immunofluorescence staining. The scale bar represents 50 μm. *D*, schematic diagram of *E*. *E*, Vero cells were seeded in 24-well plates (5 × 10^4^ cells/well) and cultured in DMEM containing 2% FBS. Hc-CATH (1.25, 2.5, and 5 μM), AC5 (2.5 μM), LL-37 (2.5 μM), or same volume of PBS (peptide solvent) was added to cells and incubated at 37 °C for 2 h. Cells were washed with PBS and incubated with ZIKV (MOI = 1). After incubation at 37 °C for 2 h, cells were washed with PBS and cultured in DMEM containing 2% FBS. After culture for 24 h, the level of AXL was tested by Western blot. Ratio of AXL to β-actin was analyzed by ImageJ. DMEM, Dulbecco’s modified Eagle’s medium; FBS, fetal bovine serum; Hc-CATH, a cathelicidin antimicrobial peptide identified from the sea snake *Hydrophis cyanocinctus*; MOI, multiplicity of infection; TIM, T-cell immunoglobulin and mucin domain.

**Figure 4:**
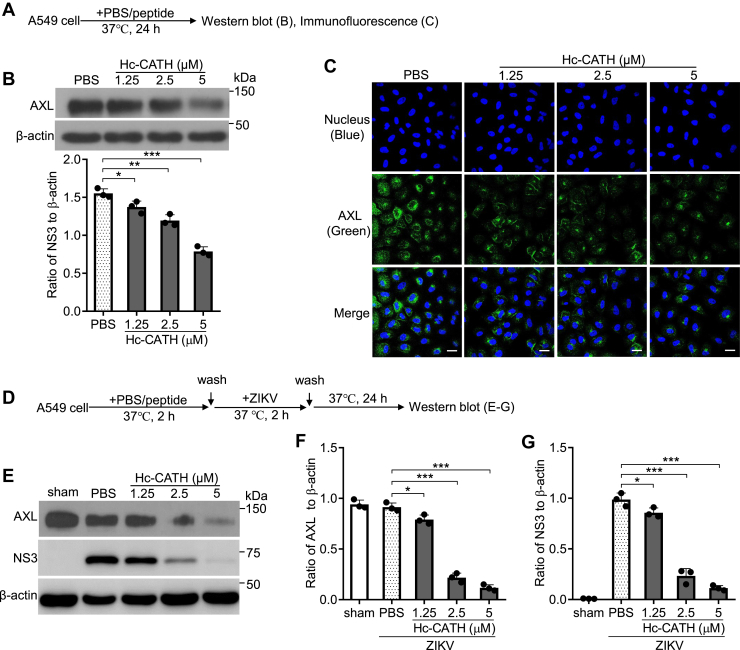
**Hc-CATH downregulates AXL in A549 cell.***A*, schematic diagram of *B* and *C*. *B*, A549 cells were seeded in 24-well plates (5 × 10^4^ cells/well) and cultured in DMEM containing 2% FBS. Hc-CATH (1.25, 2.5, and 5 μM) or same volume of PBS (peptide solvent) was added to cells and incubated at 37 °C for 24 h. The level of AXL was tested by Western blot. *C*, A549 cells were seeded in 8-well cover slip chambers (5 × 10^4^ cells/well) and cultured in DMEM containing 2% FBS. Hc-CATH (1.25, 2.5, and 5 μM) or PBS was added to cells and incubated at 37 °C for 24 h. The level of AXL was tested by immunofluorescence staining. The scale bar represents 50 μm. *D*, schematic diagram of *E* and *G*. *E* and *G*, A549 cells were seeded in 24-well plates (5 × 10^4^ cells/well) and cultured in DMEM containing 2% FBS. Hc-CATH (1.25, 2.5, and 5 μM) or PBS was added to cells and incubated at 37 °C for 2 h. Cells were washed with PBS and incubated with ZIKV (MOI = 1). After incubation at 37 °C for 2 h, cells were washed with PBS and cultured in DMEM containing 2% FBS. After culture for 24 h, AXL and ZIKV NS3 was tested by Western blot (*E*), ratio of AXL (*F*), and NS3 (G) to β-actin was analyzed by ImageJ. ∗*p* < 0.05, ∗∗*p* < 0.01, and ∗∗∗*p* < 0.001. DMEM, Dulbecco’s modified Eagle’s medium; FBS, fetal bovine serum; Hc-CATH, a cathelicidin antimicrobial peptide identified from the sea snake *Hydrophis cyanocinctus*; MOI, multiplicity of infection; ZIKV, Zika virus.

**Figure 5:**
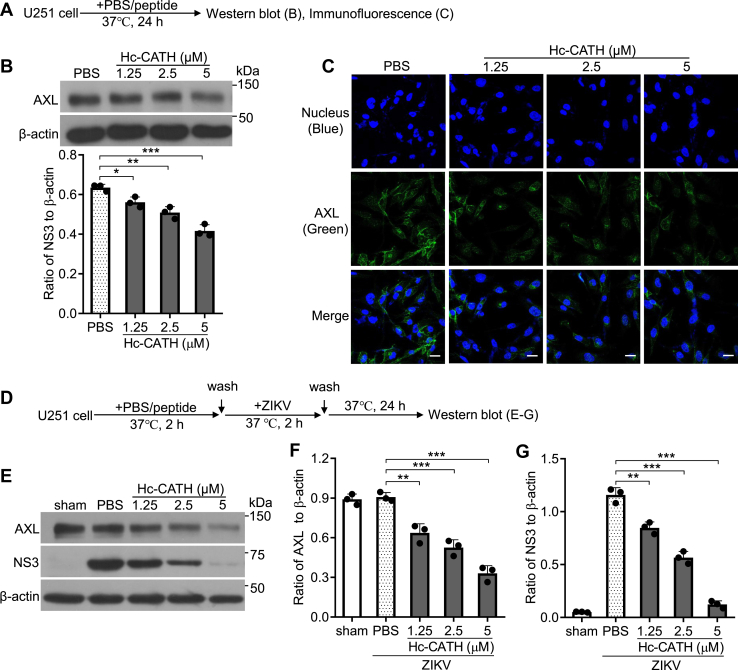
**Hc-CATH downregulates AXL in U251 cell.***A*, schematic diagram of *B* and *C*. *B*, U251 cells were seeded in 24-well plates (5 × 10^4^ cells/well) and cultured in DMEM containing 2% FBS. Hc-CATH (1.25, 2.5, and 5 μM) or same volume of PBS (peptide solvent) was added to cells and incubated at 37 °C for 24 h. The level of AXL was tested by Western blot. *C*, U251 cells were seeded in 8-well cover slip chambers (5 × 10^4^ cells/well) and cultured in DMEM containing 2% FBS. Hc-CATH (1.25, 2.5, and 5 μM) or PBS was added to cells and incubated at 37 °C for 24 h. The level of AXL was tested by immunofluorescence staining. The scale bar represents 50 μm. *D*, schematic diagram of *E*–*G*. *E*–*G*, U251 cells were seeded in 24-well plates (5 × 10^4^ cells/well) and cultured in DMEM containing 2% FBS. Hc-CATH (1.25, 2.5, and 5 μM) or PBS was added to cells and incubated at 37 °C for 2 h. Cells were washed with PBS and incubated with ZIKV (MOI = 1). After incubation at 37 °C for 2 h, cells were washed with PBS and cultured in DMEM containing 2% FBS. After culture for 24 h, AXL and ZIKV NS3 was tested by Western blot (*E*), ratio of AXL (*F*), and NS3 (G) to β-actin was analyzed by ImageJ. ∗*p* < 0.05, ∗∗*p* < 0.01, and ∗∗∗*p* < 0.001. DMEM, Dulbecco’s modified Eagle’s medium; FBS, fetal bovine serum; Hc-CATH, a cathelicidin antimicrobial peptide identified from the sea snake *Hydrophis cyanocinctus*; MOI, multiplicity of infection; ZIKV, Zika virus.

It is well known that several ZIKV entry factors, such as AXL, TIM-1, and TYRO3, can change the susceptibility of host cells to ZIKV (15). In order to identify whether Hc-CATH decreases the susceptibility of host cells by downregulating these factors, we performed experiment as indicated in Fig. 3*A*. As shown in Fig. 3*B*, Hc-CATH reduced the level of AXL in Vero cells relative to PBS after incubation for 24 h. Immunofluorescence (IF) staining confirmed that Hc-CATH did reduce the level of AXL in Vero cells after incubation for 24 h (Fig. 3*C*). While Hc-CATH did not obviously influence the other ZIKV entry factors (TYRO3 and TIM-1) at 6, 12, and 24 h post Hc-CATH addition (Fig. 3*B*). We next tested the effect of Hc-CATH on AXL in Vero cells upon ZIKV infection as indicated in Fig. 3*D*. At 24 h post Hc-CATH addition, Hc-CATH also decreased the level of AXL in Vero cells relative to PBS upon ZIKV infection (Fig. 3*E*). We then tested the effect of Hc-CATH on AXL in A549 cells and U251 cells as indicated in Fig. 4*A* and Fig. 5*A*, respectively. The results demonstrated that Hc-CATH effectively decreased the level of AXL in A549 cells (Fig. 4, *B* and *C*, E and *F*) and U251 cells (Fig. 5, *B* and *C*, *E* and *F*). Besides, Hc-CATH also decreased the susceptibility of A549 cells (Fig. 4, *E* and *G*) and U251 cells (Fig. 5, *E* and *G*) to ZIKV. The data suggest that Hc-CATH decreases the susceptibility of host cells to ZIKV by downregulating AXL.

### Cyclo-oxygenase-2/prostaglandin E2/adenylyl cyclase/PKA pathway is involved in Hc-CATH-mediated AXL downregulation

**Figure 6:**
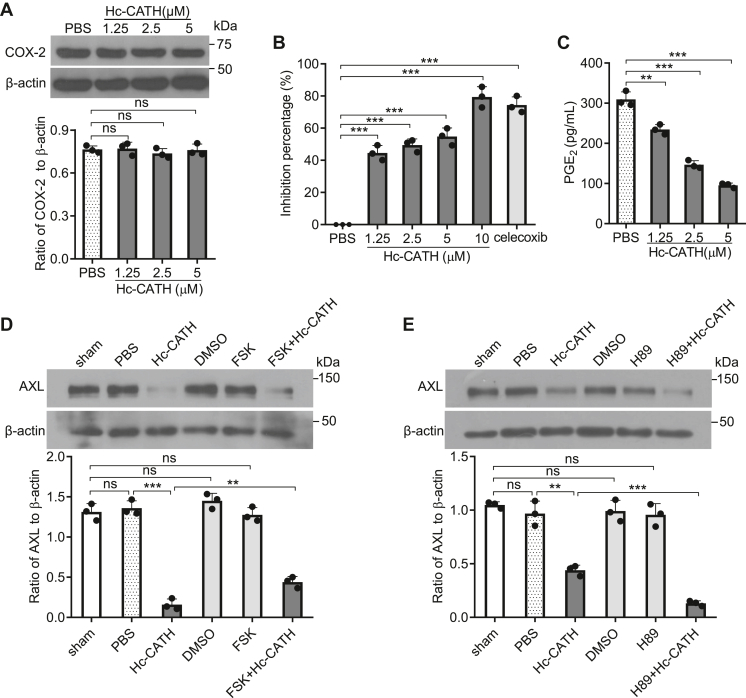
**Hc-CATH downregulates AXL *via* inhibiting COX-2/PGE**_**2**_**/AC/PKA pathway.***A*, effect of Hc-CATH on COX-2 protein level. A549 cells were incubated with Hc-CATH (1.25, 2.5, or 5 μM) or PBS (solvent of Hc-CATH) at 37 °C for 24 h, COX-2 protein level was examined by Western blot (*upper panel*) and analyzed by ImageJ (*lower panel*). *B*, effect of Hc-CATH on the enzymatic activity of COX-2. Hc-CATH (1.25, 2.5, 5, and 10 μM) or PBS was incubated with COX-2. After incubation at 37 °C for 10 min, the enzymatic activity of COX-2 was detected by enzyme activity inhibitor screening kit. Celecoxib (COX-2 inhibitor, 100 nM) was used as positive control. *C*, effect of Hc-CATH on PGE_2_ production. A549 cells were incubated with Hc-CATH (5 μM) at 37 °C. After incubation for 24 h, the level of PGE_2_ in the cell supernatant was detected by ELISA. *D* and *E*, effect of AC (*D*) and PKA (*E*) on Hc-CATH-induced AXL downregulation. A549 cells were incubated with forskolin (FSK, agonist of AC, 10 μM) or H89 (inhibitor of PKA, 5 μM) at 37 °C. After incubation for 1 h, the culture media were removed, cells were washed three times with PBS, and fresh culture media were added to cells in the presence of Hc-CATH (5 μM). After incubation for 24 h, AXL protein level was examined by Western blot (*upper panel* of *D* and *E*) and analyzed by ImageJ (*lower panel* of *D* and *E*). ns, not significant, ∗∗*p* < 0.01, and ∗∗∗*p* < 0.001. AC, adenylate cyclase; COX-2, cyclo-oxygenase-2; Hc-CATH, a cathelicidin antimicrobial peptide identified from the sea snake *Hydrophis cyanocinctus*; PGE_2_, prostaglandin E_2._

As described previously, cycloo-xygenase-2 (COX-2) can regulate the synthesis of prostaglandin E_2_ (PGE_2_), and PGE_2_ can activate the expression of downstream signal molecules that make AXL stably express on cell surface (38). We were interested to explore whether this signaling pathway is involved in Hc-CATH-mediated AXL downregulation. We found that Hc-CATH did not influence the expression of COX-2 (Fig. 6*A*), but it significantly inhibited the enzyme activity of COX-2 in a dose-dependent manner (Fig. 6*B*). At concentration of 10 μM, the inhibitory rate of Hc-CATH against COX-2 activity could reach about 80%. As expected, the addition of Hc-CATH significantly reduced the synthesis of PGE_2_ in a dose-dependent manner (Fig. 6*C*). It was reported that PGE_2_ can lead to the activation of adenylyl cyclase (AC) and an increase in the cAMP. Subsequently, cAMP can activate PKA (39). To see if these downstream signal molecules are involved in Hc-CATH-induced AXL downregulation, we added forskolin (FSK, a specific activator of AC) to the cell culture. As shown in Fig. 6*D*, the addition of FSK effectively counteracted the Hc-CATH-induced AXL downregulation. However, a specific PKA kinase inhibitor, H89, significantly facilitated Hc-CATH-mediated AXL downregulation (Fig. 6*E*). These results indicate that COX-2/PGE_2_/AC/PKA pathway is involved in Hc-CATH-mediated AXL downregulation.

### Hc-CATH-induced AXL downregulation reverses the negative regulation of AXL on type I IFN signaling

**Figure 7:**
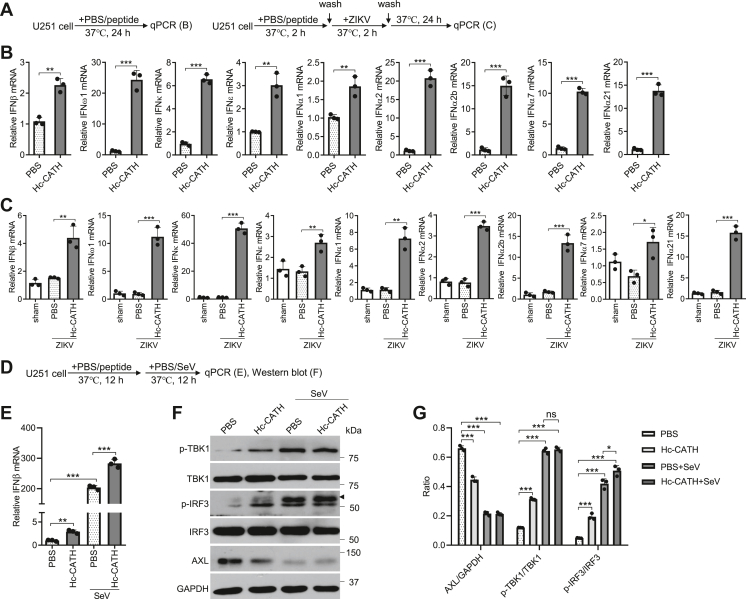
**Hc-CATH-induced AXL downregulation reverses the negative regulation of AXL on type I IFN signaling.***A*, schematic diagram of *B* and *C*. *B*, effect of Hc-CATH on type I IFN signaling. U251 cells were cultured in the presence of Hc-CATH (2.5 μM) or same volume of PBS (solvent of peptide). After culture at 37 °C for 24 h, type I IFN genes were tested by qPCR. *C*, effect of Hc-CATH on type I IFN signaling upon ZIKV challenge. U251 cells were cultured in the presence of Hc-CATH (2.5 μM) or PBS (solvent of peptide) at 37 °C for 2 h. Cells were washed with PBS and incubated with ZIKV (MOI = 1) at 37 °C for 2 h. Cell were washed with PBS again and cultured in DMEM containing 2% FBS at 37 °C for 24 h. Type I IFN genes were tested by qPCR. *D*, schematic diagram of *E*–*G*. *E* and *F*, U251 cells were cultured in the presence of Hc-CATH (2.5 μM) or PBS (solvent of peptide) at 37 °C for 12 h. Cells were stimulated with SeV (MOI = 1) at 37 °C for 12 h. Type I IFN gene was tested by qPCR (*E*). Type I IFN protein and AXL were tested by Western bolt (*F*), and the ratio was analyzed by ImageJ (*G*). ns, not significant, ∗*p* < 0.05, ∗∗*p* < 0.01, and ∗∗∗*p* < 0.001. DMEM, Dulbecco’s modified Eagle’s medium; FBS, fetal bovine serum; IFN, interferon; MOI, multiplicity of infection; qPCR, quantitative PCR; SeV, Sendai virus; ZIKV, Zika virus.

Several studies have shown that AXL is an important negative regulator of type I IFN signaling and facilitate ZIKV infection by antagonizing type I IFN (40). We next tested whether Hc-CATH-induced AXL downregulation reverses the negative regulation of AXL on type I IFN as indicated (Fig. 7*A*). We found that Hc-CATH significantly increased the levels of type I IFN genes (Fig. 7*B*). Meanwhile, Hc-CATH also upregulated the levels of type I IFN genes in response to ZIKV infection (Fig. 7*C*). In contrast, type I IFN genes were only slightly activated or, in some cases, downregulated in response to ZIKV infection without Hc-CATH treatment (Fig. 7*C*). As described previously, ZIKV has evolved multiple molecular mechanisms to escape type I IFN production (41). Next, we used Sendai virus (SeV) to amplify type I IFN signaling (42) and verified the effect of Hc-CATH on type I IFN response upon SeV stimulation as indicated in Fig. 7*D*. We found that SeV markedly induced IFN-β expression, and Hc-CATH treatment significantly increased IFN-β expression in response to SeV stimulation as compared with PBS treatment (Fig. 7*E*). To further explore the role of Hc-CATH in type I IFN response, we investigated its effect on IFN signaling pathway by detecting the phosphorylation of TANK-binding kinase 1 and interferon regulatory factor 3 (IRF3) in the presence or the absence of SeV stimulation. As shown in Fig. 7, *F* and *G*, Hc-CATH increased the phosphorylation of TANK-binding kinase 1 and IRF3 relative to PBS, and it also enhanced the phosphorylation of IRF3 (indicated by a triangle) in response to SeV stimulation. The data suggest that Hc-CATH-induced AXL downregulation reverses the negative regulatory effect of AXL on type I IFN response.

### Hc-CATH directly inactivates ZIKV particles by disrupting viral membrane

**Figure 8:**
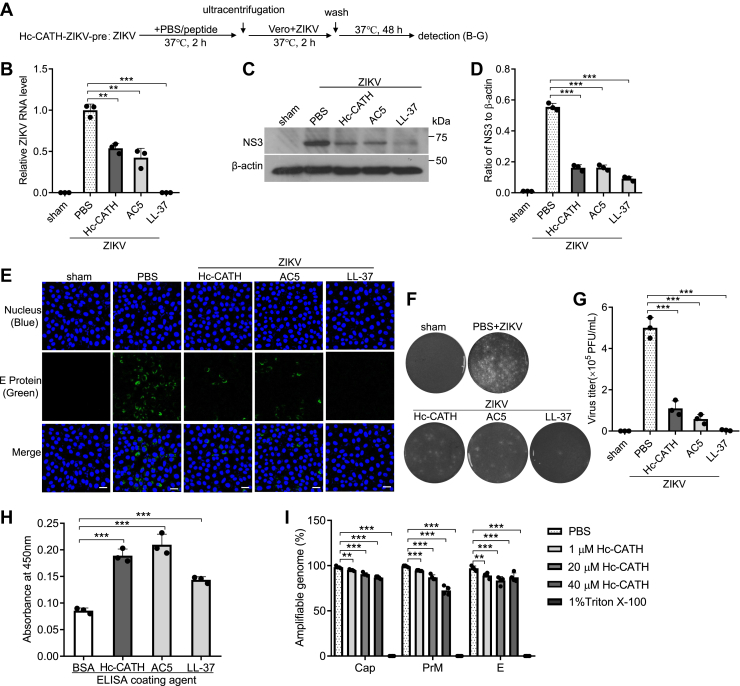
**Hc-CATH directly inactivates ZIKV particles by disrupting viral membrane.***A*, schematic diagram of *B*–*G* (Hc-CATH-Vero-pre). *B*–*G*, direct inactivation of ZIKV by Hc-CATH. ZIKVs (MOI = 1) were incubated with Hc-CATH (2.5 μM), AC5 (2.5 μM), LL-37 (2.5 μM), or PBS (peptide solvent) at 37 °C for 2 h, and then the ZIKV–PBS mixture and ZIKV–peptide mixture were centrifugated at 100,000*g* for 70 min. The pellets were washed with PBS and centrifugated at 100,000*g* for 70 min again. The pellets were resuspended in PBS, added to Vero cells, and incubated for 2 h. Cells were washed with PBS and cultured in fresh DMEM containing 2% FBS. After culture at 37 °C for 48 h, the intracellular ZIKV RNA (*B*), NS3 protein (*C* and *D*), E protein level (*E*, the scale bar represents 50 μm), and extracellular ZIKV titer (*F* and *G*) were tested by qPCR, Western blot, immunofluorescence staining, and plaque-forming assay, respectively. The raw data files used in the creation of *F* are presented in Fig. S5. *H*, binding of Hc-CATH to ZIKV. High-affinity binding plates were coated with 0.25 μM of Hc-CATH, BSA, AC5, or LL-37. Then, 1 × 10^6^ PFU of ZIKV was added and incubated. Wells were exposed to anti-ZIKV E protein antibody, HRP-labeled secondary antibody, and TMB substrate in turn. Absorbance at 450 nm was measured after reactions were stopped using 0.5 M sulfuric acid. *I*, leakage of ZIKV genomic RNA induced by Hc-CATH. ZIKV (about 10^5^ PFU) was incubated with Hc-CATH (1, 20, and 40 μM), PBS (solvent of peptide), or 1% Triton X-100 (positive control) at 37 °C for 2 h. The released genomic RNA was digested by micrococcal nuclease at 37 °C for 4 h. After the residual RNase was inactivated, the undigested genomic RNA in the intact ZIKV particles was extracted and quantified by testing the genes of PrM, E, and Cap proteins, respectively. ∗∗*p* < 0.01 and ∗∗∗*p* < 0.001. BSA, bovine serum albumin; FBS, fetal bovine serum; Hc-CATH, a cathelicidin antimicrobial peptide identified from the sea snake *Hydrophis cyanocinctus*; HRP, horseradish peroxidase; MOI, multiplicity of infection; PFU, plaque-forming unit; qPCR, quantitative PCR; ZIKV, Zika virus.

It was reported that some anti-ZIKV peptides can directly inactivate virions by disrupting virus particles (28, 43). To investigate whether Hc-CATH exhibited direct effect on ZIKV, ZIKV virions were incubated with PBS or peptide at 37 °C for 2 h, and the infectivity of ZIKV virions was tested as indicated in Fig. 8*A* (Hc-CATH-virus-pre). As shown in Fig. 8, *B*–*G*, the incubation of ZIKV with Hc-CATH significantly decreased the intracellular ZIKV RNA (Fig. 8*B*), NS3 protein (Fig. 8, *C* and *D*), E protein (Fig. 8*E*), and extracellular ZIKV titer (Fig. 8, *F* and *G*) relative to PBS incubation, indicating that the incubation of Hc-CATH with ZIKV directly inactivated viral particles. To further determine the interaction of Hc-CATH with ZIKV, we tested the binding of Hc-CATH to ZIKV by ELISA. As shown in Fig. 8*H*, Hc-CATH had a stronger binding affinity with ZIKV as compared with bovine serum albumin, which proved that Hc-CATH could directly bind to ZIKV particles. We next explored whether Hc-CATH disrupt ZIKV envelope by testing the potential release of viral genomic RNA from ZIKV after the incubation of ZIKV with Hc-CATH. As shown in Fig. 8*I*, the genomic RNA of PBS-treated virions was protected from digestion by micrococcal nuclease, which was determined as 100%. However, the genomic RNA of Hc-CATH-treated virions was digested by micrococcal nuclease. These data suggest that Hc-CATH can bind to ZIKV, disrupt viral membrane, induce the leakage of viral genomic RNA, and then inactivate ZIKV virions.

### Hc-CATH does not act on the late stage of ZIKV infection

**Figure 9:**
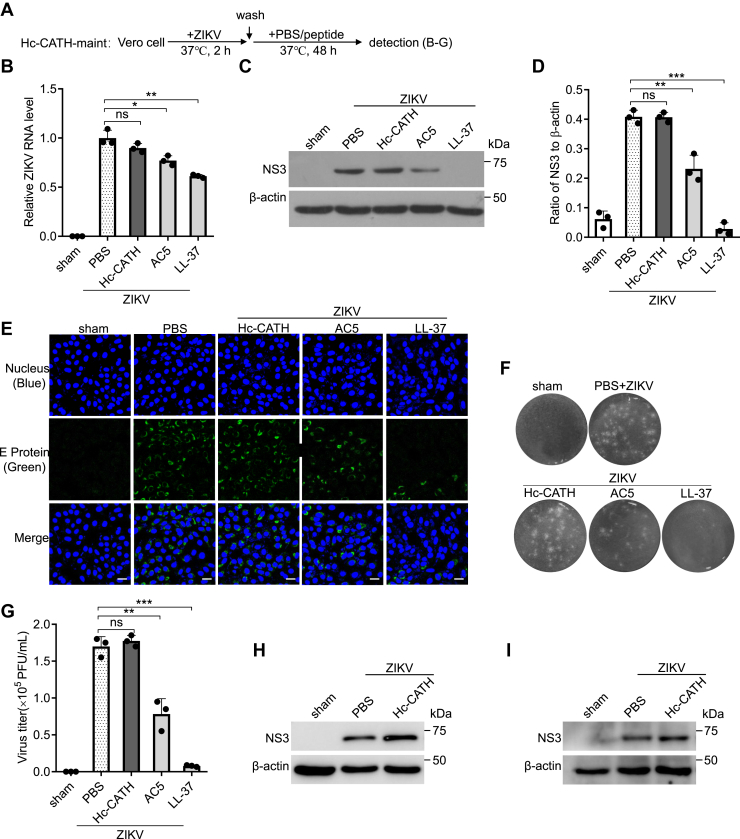
**Hc-CATH does not act on ZIKV replication stage.***A*, schematic diagram of *B*–*I* (Hc-CATH-maint). *B*–*I*, Vero (*B*–*G*), A549 (*H*), or U251 (*I*) cells were incubated with ZIKV (MOI = 1) at 37 °C for 2 h, washed with PBS, and transferred with fresh DMEM containing 2% FBS and Hc-CATH (2.5 μM), AC5 (2.5 μM), LL-37 (2.5 μM), or PBS (solvent of peptide). After culture at 37 °C for 48 h, intracellular ZIKV RNA (*B*), NS3 protein (*C*, *D*, *H*, and *I*), E protein level (*E*, the scale bar represents 50 μm), and extracellular ZIKV titer (*F* and *G*) were detected by qPCR, Western blot, immunofluorescence staining, and plaque-forming assay, respectively. The raw data files used in the creation of *F* are presented in Fig. S6. ns, not significant, ∗*p* < 0.05, ∗∗*p* < 0.01, and ∗∗∗*p* < 0.001. DMEM, Dulbecco’s modified Eagle’s medium; FBS, fetal bovine serum; Hc-CATH, a cathelicidin antimicrobial peptide identified from the sea snake *Hydrophis cyanocinctus*; MOI, multiplicity of infection; qPCR, quantitative PCR; ZIKV, Zika virus.

To see if Hc-CATH act on ZIKV replication stage, we conducted experiments as indicated in Fig. 9*A* (Hc-CATH-maint). When Hc-CATH was maintained in the cell culture after ZIKV infection, Hc-CATH did not reduce the intracellular ZIKV RNA (Fig. 9*B*), NS3 protein (Fig. 9, *C* and *D*), E protein (Fig. 9*E*), and extracellular ZIKV titer (Fig. 9, *F* and *G*) in Vero cells relative to PBS. Hc-CATH also did not inhibit ZIKV infection in A549 cells (Fig. 9*H*) and U251 cells (Fig. 9*I*) when Hc-CATH was added to the cell culture after ZIKV infection. The results indicate that Hc-CATH does not act on the late stage of ZIKV replication.

### Helix and phenylalanine residues are key structural requirements for Hc-CATH against ZIKV infection

**Figure 10:**
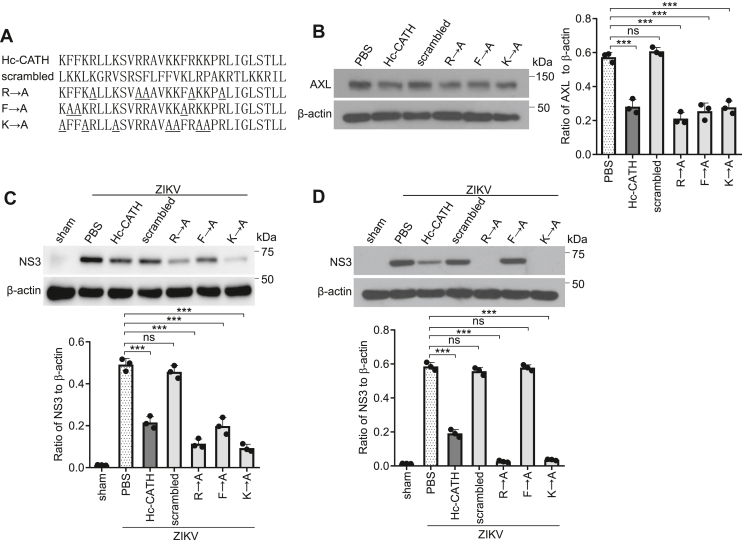
**Helix and phenylalanine residues are key structural requirements for Hc-CATH against ZIKV infection.***A*, Hc-CATH and its mutant peptides. *B*, downregulation of AXL by Hc-CATH and its mutant peptides. *Left panel*, immunoblots. *Right panel*, ratio of AXL to β-actin analyzed by ImageJ. *C*, decrease of the susceptibility of Vero cells to ZIKV. *Upper panel*, immunoblots. *Lower panel*, ratio of NS3 to β-actin analyzed by ImageJ. *D*, inactivation of ZIKV by Hc-CATH and its mutant peptides. *Upper panel*, immunoblots. *Lower panel*, ratio of NS3 to β-actin analyzed by ImageJ. The α-helix of Hc-CATH was disrupted by scrambling the amino acid sequence, and the arginine, lysine, or phenylalanine residues of Hc-CATH were substituted with alanine residues, respectively. Downregulation of AXL, decrease of the susceptibility of Vero cells to ZIKV, and inactivation of ZIKV by Hc-CATH and its mutant peptides were assayed. ns, not significant, ∗∗∗*p* < 0.001. Hc-CATH, a cathelicidin antimicrobial peptide identified from the sea snake *Hydrophis cyanocinctus*; ZIKV, Zika virus.

In our previous study, Hc-CATH was demonstrated to have helical structure, positively charged residues (five arginine and seven lysine residues), and aromatic residues (three phenylalanine residues) (26). In order to understand the key structural requirements of Hc-CATH against ZIKV infection, the helix of Hc-CATH was disrupted by scrambling the amino acid sequence (Fig. 10*A* and S2), and the arginine, lysine, or phenylalanine residues were substituted with alanine residues (Fig. 10*A*), respectively. We found that Hc-CATH was unable to downregulate AXL (Fig. 10*B*) and decrease the susceptibility of Vero cells to ZIKV (Fig. 10*C*) when the helix was disrupted. Besides, Hc-CATH was unable to directly inactivate ZIKV when the helix was disrupted, or phenylalanine residues were substituted with alanine residues (Fig. 10*D*). The data indicate that α-helix and phenylalanine residues are key structural requirements for Hc-CATH against ZIKV infection.

### Hc-CATH shows preventive and therapeutic effect against ZIKV infection in mice

**Figure 11:**
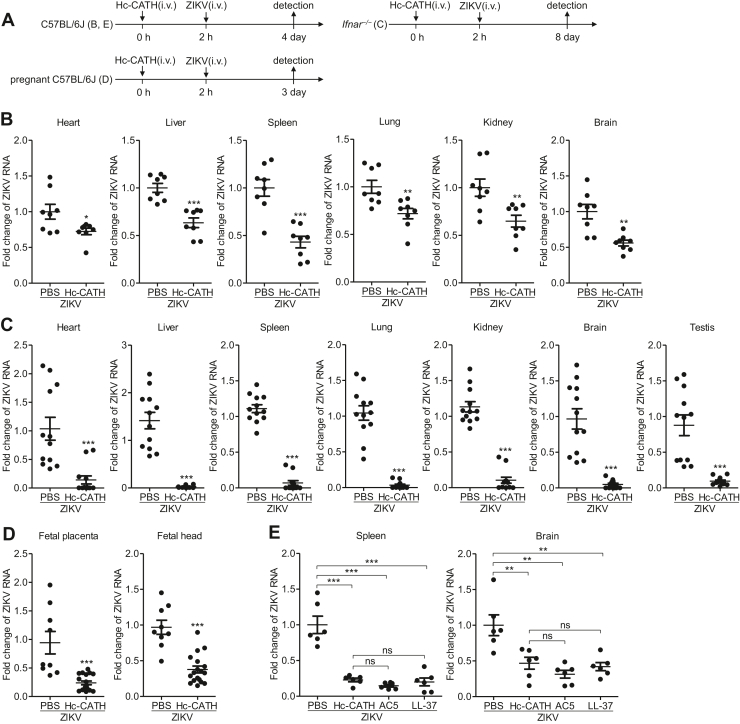
**Hc-CATH shows prophylactic efficacy against ZIKV infection in mice.***A*, schematic diagram of *B*–*E*. *B*–*E*, C57BL/6J mice (*B* and *E*), *Ifnar1*^−/−^ mice (*C*), and pregnant C57BL/6J mice (*D*) were intravenously administrated with peptide at 2 h before intravenous injection of ZIKV (10^6^ PFU/mouse). At 4, 8, and 3 days post ZIKV inoculation, C57BL/6J mice, *Ifnar1*^−/−^ mice, and pregnant C57BL/6J mice were sacrificed, respectively. Tissues and fetal mice were collected for testing ZIKV RNA by qPCR. ns, not significant, ∗*p* < 0.05, ∗∗*p* < 0.01, and ∗∗∗*p* < 0.001. Hc-CATH, a cathelicidin antimicrobial peptide identified from the sea snake *Hydrophis cyanocinctus*; PFU, plaque-forming unit; qPCR, quantitative PCR; ZIKV, Zika virus.

Since Hc-CATH effectively inhibits ZIKV infection *in vitro*, we were interested to test whether Hc-CATH can resist ZIKV infection *in vivo*. We first evaluated whether Hc-CATH has preventive efficacy against ZIKV infection. C57BL/6J mice, IFNα/β receptor–deficient (*Ifnar1*^−/−^) mice, and pregnant C57BL/6J mice were intravenously administrated with Hc-CATH at 2 h before intravenous injection of ZIKV as indicated in Fig. 11*A*. As shown in Fig. 11, *B*–*D*, intravenous injection of Hc-CATH at 2 h before ZIKV infection significantly decreased ZIKV replication in C57BL/6J mice (Fig. 11*B*), *Ifnar1*^−/−^ mice (Fig. 11*C*), fetal placenta, and fetal mice (Fig. 11*D*), implying that intravenous injection of Hc-CATH at 2 h before ZIKV challenge provides prophylactic efficacy against ZIKV infection in mice.

**Figure 12:**
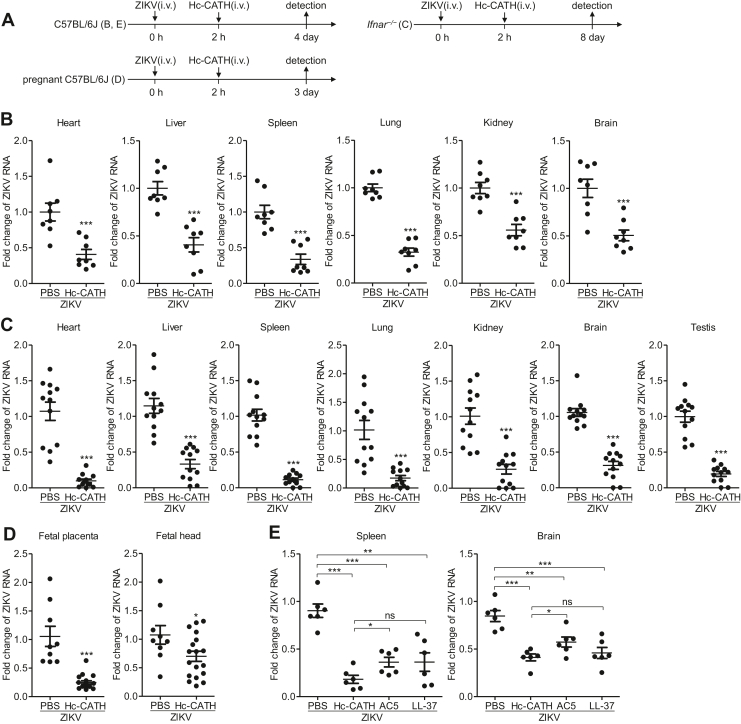
**Hc-CATH shows therapeutic efficacy against ZIKV infection in mice.***A*, schematic diagram of *B*–*E*. *B*–*E*, C57BL/6J mice (*B* and *E*), *Ifnar1*^−/−^ mice (*C*), and pregnant C57BL/6J mice (*D*) were intravenously administrated with peptide at 2 h after intravenous injection of ZIKV (10^6^ PFU/mouse). At 4, 8, and 3 days post ZIKV inoculation, C57BL/6J mice, *Ifnar1*^−/−^ mice, and pregnant C57BL/6J mice were sacrificed, respectively. Tissues and fetal mice were collected for testing ZIKV RNA by qPCR. ns, not significant, ∗*p* < 0.05, ∗∗*p* < 0.01, and ∗∗∗*p* < 0.001. Hc-CATH, a cathelicidin antimicrobial peptide identified from the sea snake *Hydrophis cyanocinctus*; PFU, plaque-forming unit; qPCR, quantitative PCR; ZIKV, Zika virus.

We then evaluated whether Hc-CATH has therapeutic effect against ZIKV infection. C57BL/6J mice, *Ifnar1*^−/−^ mice, and pregnant C57BL/6J mice were intravenously administrated with Hc-CATH at 2 h after intravenous injection of ZIKV as indicated in Fig. 12*A*. As shown in Fig. 12, *B*–*D*, intravenous injection of Hc-CATH at 2 h after ZIKV infection significantly attenuated ZIKV replication in C57BL/6J mice (Fig. 12*B*), *Ifnar1*^−/−^ mice (Fig. 12*C*), fetal placenta, and fetal mice (Fig. 12*D*), suggesting that intravenous injection of Hc-CATH at 2 h after ZIKV challenge provides therapeutic efficacy against ZIKV infection in mice. In addition, the anti-ZIKV efficacy of Hc-CATH seemed to be weaker than that of AC5 and LL-37 *in vitro* (Fig. 1), but its anti-ZIKV efficacy was comparable with or even better than that of AC5 and LL-37 *in vivo* (Figs. 11*E* and 12*E*).

## Discussion

It has been shown that AMPs (also called host defense peptides) not only have antibacterial and immunomodulatory activities but also can be used as an important source of antiviral drug development with broad antiviral spectrum (28, 29, 44, 45). In recent years, a series of AMPs/host defensive peptides were shown to confer protection against ZIKV infection, including human cathelicidin AMP (46) and defensin AMP (47), bovine cathelicidin AMP (46), scorpion venom peptides derived from *Scorpio maurus palmatus* (48) and *Euscorpiops validus* (49), spider venom peptide from *Alopecosa nagpag* (50), frog host defense peptide from *Indosylvirana aurantiaca* (51), and snail antibacterial peptide from *Pomacea poeyana* (52). We herein found that the snake venom–derived cathelicidin AMP (Hc-CATH) from *H. cyanocinctus* exhibited potent preventive and therapeutic efficacy against ZIKV infection *in vitro* and *in vivo*, which provides a novel anti-ZIKV peptide isolated from AMPs/hose defense peptides. Compared with the antiviral drugs based on small molecular compounds and antibodies, peptide-based antiviral drugs have attracted more and more attention because of their good safety and lower development cost and better safety (43, 53).

Anti-ZIKV peptides from biological sources display multiple antiviral mechanisms against ZIKV infection. Human cathelicidin LL-37 and its derived peptide GF-17, mouse cathelicidin CRAMP, bovine cathelicidin–derived peptide BMAP-18, *Bungarus fasciatus* cathelicidin-derived peptide ZY13, host defense peptide Yodha from *I. aurantiaca*, and Z2 from the stem region of ZIKV envelope protein are virucidal to ZIKV by disrupting the integrity of the viral membrane (28, 43, 46, 51, 54), which can directly inactivate ZIKV virion and comprise the major antiviral mechanism against ZIKV infection. Human cathelicidin–derived peptide GF-17, bovine cathelicidin–derived peptide BMAP-18, *B. fasciatus* cathelicidin–derived peptide ZY13, and scorpion venom peptide Smp76 can inhibit ZIKV infection by enhancing type I IFN response (46, 48, 54). Scorpion venom peptide Ev37 from scorpion *E. validus* can alkalize acidic organelles to prevent low pH-dependent fusion of the viral membrane–endosomal membrane (49). Spider venom peptide An1a from *A. nagpag* restricts ZIKV infection by inhibiting NS2B–NS3 protease (50).

Hc-CATH can disrupt ZIKV particle, induce the leakage of genomic RNA, and finally inactivate virion. The direct inactivation of ZIKV by Hc-CATH is reminiscent of the mechanism of action of other biological peptides like LL-37 (28), CRAMP (28), GF-17 (46), BMAP-18 (46), ZY13 (54), Yodha (51), and Z2 (43), which exhibit the same anti-ZIKV mechanism as that of Hc-CATH. AMPs usually form helical structure that has both hydrophobic and amphiphilic domains, which can interact with lipid membranes, resulting in instability, translocation, pore formation, or cleavage of lipid membranes (43). Therefore, viral envelope can be an important target for AMPs. To verify whether such structure is essential for the direct inactivation of ZIKV by Hc-CATH, the helix of Hc-CATH was disrupted by scrambling the amino acid sequence. We found that scrambled Hc-CATH did not directly inactivate ZIKV virion, suggesting that the helical structure is a key structural requirement for the direct inactivation of ZIKV by Hc-CATH. In addition to helical structure, aromatic residue (phenylalanine) was also shown to be critical for the direct inactivation of ZIKV by Hc-CATH. Our previous studies have shown that helical structure and aromatic residue are essential for the AMPs to directly kill bacteria by disrupting bacterial membrane (26, 55). These indicate that helical structure and aromatic residues may be common structural requirements for inactivating enveloped virus and bacteria by disrupting microbial membrane. However, the substitution of positively charged residues with alanine does not inhibit the Hc-CATH-mediated direct inactivation of ZIKV. While the substitution of arginine/lysine with alanine markedly attenuates the direct antibacterial activity of Hc-CATH (26).

Intriguingly, Hc-CATH effectively downregulates AXL in host cells. AXL is a member of TAM receptors that play an important role in ZIKV infection (22, 40). AXL exhibits dual role during ZIKV infection. On the one hand, AXL acts as a cofactor that mediates ZIKV entry (40). TAM receptors can recognize PS. Similar to other flaviviruses, the envelope of ZIKV contains PS, which facilitates the adsorption and internalization of TAM receptors. As described previously, AXL can promote ZIKV entry in human Sertoli cells (25) and human skin cells (11). On the other hand, AXL promotes ZIKV infection by antagonizing type I IFN signaling (22, 40). AXL plays a pivotal role in maintaining the immunosuppressive milieu of the testis, enhancing ZIKV infection by negatively regulating antiviral immune response (25). Consistent with these findings, Hc-CATH-mediated AXL downregulation effectively decreases the susceptibility of host cells to ZIKV and reverses the negative regulation of AXL on type I IFN signaling. In addition, COX-2//PGE_2_/AC/PKA pathway is involved in Hc-CATH-mediated AXL downregulation. Our findings reveal another possible mechanism of biological peptides against ZIKV infection, providing new insight into the mechanism of action of anti-ZIKV peptides isolated from AMPs/host defense peptides.

Innate immune response is the first line for host defense against invading viruses. In general, the innate immune response is initiated by identifying pathogen-associated molecular patterns accumulated during infection. Pathogen-associated molecular patterns bind to pattern recognition receptors in the nucleus or in the cytoplasm, resulting in the activation of intracellular cascade signals and the upregulation of a variety of innate immune molecules and cytokines (56). Type I IFNs are important cytokines for host defense against viruses. They are responsible for coordinating the antiviral state in infected cells and adjacent cells and controlling virus infection by guiding the activation or transportation of immune cells (41). Our findings have shown that Hc-CATH can reverse the negative regulation of AXL on type I IFN response, which seems to be an important antiviral mechanism of Hc-CATH against ZIKV infection. However, Hc-CATH still possesses strong antiviral efficacy against ZIKV in *Ifnar1*^−/−^ mice, implying that the anti-ZIKV effect of Hc-CATH does not depend on the production of type I IFNs induced by Hc-CATH. It is more likely that Hc-CATH has multiple antiviral mechanism, including direct inactivation of ZIKV, induction of AXL downregulation, and enhancement of type I IFN response, and the protective efficacy of Hc-CATH in *Ifnar1*^−/−^ mice may be attributed to Hc-CATH-mediated ZIKV inactivation and AXL downregulation. In this study, we claimed that Hc-CATH enhanced type I IFN response of host cells by downregulating AXL, but we cannot exclude the direct regulatory effect of Hc-CATH on type I IFN response of host cells, which need to further elucidated in future.

In summary, Hc-CATH, cathelicidin AMP identified from sea snake *H. cyanocinctus*, was shown to inhibit ZIKV infection *in vitro* and provide preventive and therapeutic efficacy against ZIKV infection *in vivo*. Intriguingly, Hc-CATH downregulated AXL in host cells, thus decreased the susceptibility of host cells to ZIKV. Hc-CATH also acted as an inactivator of ZIKV by disrupting viral membrane and inducing the leakage of genomic RNA. Helical structure and phenylalanine residues are key structural requirements for Hc-CATH against ZIKV infection. Our findings provide a new peptide candidate for anti-ZIKV drug development and new insight into the cathelicidin AMPs against ZIKV infection.

## Experimental procedures

### Key resource table

**Table:** 

Reagent type (species) or resource	Designation	Source/reference	Identifiers	Additional information
Cell line (*Homo sapiens*)	A549	National Collection of Authenticated Cell Cultures	CSTR: 19375.09.3101HUMSCSP503	https://www.cellbank.org.cn/
Cell line (*Homo sapiens*)	U251	National Collection of Authenticated Cell Cultures	CSTR: 19375.09.3101HUMTCHu58	https://www.cellbank.org.cn/
Cell line (*Chlorocebus aethiops*)	Vero	National Collection of Authenticated Cell Cultures	CSTR: 19375.09.3101MONSCSP520	Cells were identified by flow cytometry
Commercial assay or kit	Cell Counting Kit-8	Dojindo	Catalog no.: CK04-500T	
Commercial assay or kit	Human PGE_2_ ELISA Kit	Nanjing Jiancheng Bioengineering Institute	Catalog no.: H099-1	
Commercial assay or kit	Cyclo-oxygenase 2 Inhibitor Screening Kit	Beyotime Biotechnology	Catalog no.: S0168	
Commercial assay or kit	Trizol reagent	Life Technologies	Catalog no.: 15596018	
Commercial assay or kit	PrimeScript RT reagent kit	Takara	Catalog no.: RR037A	
Antibody	Rabbit polyclonal anti-ZIKV NS3	Gene Tex	Catalog no.: GTX133309	WB (1:2000 dilution)
Antibody	Rabbit polyclonal anti-ZIKV E	Gene Tex	Catalog no.: GTX133314	IF (1:500 dilution)
Antibody	Rabbit monoclonal anti-TYRO3	abcam	Catalog no.: ab109231	WB (1:1000 dilution)
Antibody	Rabbit polyclonal anti-TIM-1	abcam	Catalog no.: ab47635	WB (1:1000 dilution)
Antibody	Rabbit monoclonal anti-AXL	Cell Signaling Technology	Catalog no.: 8661S	WB (1:1000 dilution)
Antibody	Rabbit monoclonal anti–TANK-binding kinase 1	Cell Signaling Technology	Catalog no.: 38066S	WB (1:1000 dilution)
Antibody	Rabbit monoclonal anti-Phospho-TANK-binding kinase 1	Cell Signaling Technology	Catalog no.: 5483S	WB (1:1000 dilution)
Antibody	Rabbit monoclonal anti-IRF3	Cell Signaling Technology	Catalog no.: 11904S	WB (1:1000 dilution)
Antibody	Rabbit monoclonal anti-Phospho-IRF3	Cell Signaling Technology	Catalog no.: 29047S	WB (1:1000 dilution)
Antibody	Rabbit monoclonal anti-COX-2	Beyotime Biotechnology	Catalog no.: AF1924	WB (1:200 dilution)
Antibody	Mouse monoclonal anti-β-actin	Abmart	Catalog no.: T40104	WB (1:5000 dilution)
Chemical compound, drug	FSK	Beyotime Biotechnology	Catalog no.: S1612	
Chemical compound, drug	H89	Beyotime Biotechnology	Catalog no.: S1643	

Abbreviation: WB, Western blot.

### Cell, virus, and peptide

African green monkey kidney epithelial cell line (Vero E6 cells), human pulmonary epithelial cell line (A549 cells), and human astrocyte cell line (U251 cells) were grown and maintained in Dulbecco’s modified Eagle’s medium (DMEM) supplemented with 10% heat-inactivated fetal bovine serum (FBS) and antibiotics (100 U/ml penicillin and 100 mg/ml streptomycin) at 37 °C with 5% CO_2_. Fluorescent quantitative PCR (qPCR; forward primer, 5′-GGGAGCAAACAGGATTAGATACCCT-3′, reverse primer, 5′-TGCACCATCTGTCACTCTGTTAACCTC-3′) was performed to confirm that the cell lines were negative for mycoplasma contamination.

ZIKV strain PRVABC59 (GenBank number: KU501215) was originally obtained from Dr Feifei Yin at Hainan Medical College. ZIKV was propagated in Vero cells and stored at −80 °C. The virus titer was determined in Vero cells using plaque-forming assay.

Synthetic peptides, including Hc-CATH, LL-37 (positive peptide, human cathelicidin), and AC5 (positive peptide, anti-ZIKV peptide from *Aedes aegypti*), are listed in Table S1 and purchased from SynPeptide Co Ltd. The crude peptide was purified by reversed-phase high performance liquid chromatography and analyzed by mass spectrometry to confirm the purity higher than 98%.

### Mice

C57BL/6J mice, pregnant C57BL/6J mice, and *Ifnar1*^−/−^ mice were housed in specific pathogen-free conditions. The C57BL/6J mice and pregnant C57BL/6J mice were ordered from Shanghai SLAC Laboratory Animal Co Ltd. *Ifnar1*^−/−^ mice were gifted by Dr Chunsheng Dong’s laboratory at Soochow University and bred in our laboratory. Animal experiments were performed in accordance with the Institutional Animal Care and Use Committee of Soochow University, and all research protocols were approved by the Animal Ethical Committee of Soochow University. All surgeries of mice were performed under pentobarbital sodium anesthesia with minimum fear, anxiety, and pain.

### Antiviral activity of Hc-CATH against ZIKV *in vitro*

Vero cells were seeded in 24-well plates (5 × 10^4^ cells/well). After cells were adhered to plates, cells were incubated with ZIKV (multiplicity of infection [MOI] = 1) in the presence of noncytotoxic concentration of Hc-CATH, AC5 (positive control), LL-37 (positive control), or PBS (solvent of peptide). After incubation at 37 °C for 2 h, the culture media were removed, cells were washed with PBS and transferred with fresh culture media containing 2% FBS, and Hc-CATH, AC5, LL-37, or PBS was supplemented to each well. After incubation at 37 °C for 48 h, cytopathic effect was observed by microscopy, and Cell Counting Kit-8 was applied to determine the percent of cytopathic effect. The percent of cytopathic effect was calculated as (A_sham_ –A_treatment_)/A_sham_ × 100 (where A_sham_ represents the absorbance of uninfected cells, A_treatment_ represents the absorbance of PBS- or peptide-treated cells post ZIKV infection). The intracellular ZIKV RNA, NS3 protein, E protein, and extracellular ZIKV titer were tested by qPCR, WB, IF, and plaque-forming assay, respectively (28). Primers used for qPCR are listed in Table S2.

### Effect of Hc-CATH on the susceptibility of host cells to ZIKV

Vero, A549, or U251 cells were incubated with peptide or PBS at 37 °C for 2 h. The cells were washed three times with PBS and incubated with ZIKV (MOI = 1) at 37 °C for 2 h. The culture media were replaced with fresh DMEM containing 2% FBS. After incubation at 37 °C for 48 h, intracellular ZIKV RNA, NS3 protein, E protein, and extracellular ZIKV titer were detected by qPCR, WB, IF, and plaque-forming assay, respectively (28).

### Effect of Hc-CATH on ZIKV entry factors in host cells

Vero cells were incubated with Hc-CATH (1.25, 2.5, and 5 μM), AC5 (2.5 μM), LL-37 (2.5 μM), or PBS (solvent of peptide). After incubation at 37 °C for 6, 12, or 24 h, the cells were lysed with radioimmunoprecipitation assay lysis buffer after washing with PBS. The levels of ZIKV entry factors, including AXL, TYRO3, and TIM-1, were detected by WB analysis. The level of AXL was confirmed by IF after Vero cells were incubated with Hc-CATH (1.25, 2.5, and 5 μM), AC5 (2.5 μM), LL-37 (2.5 μM), or PBS at 37 °C for 24 h.

The effect of Hc-CATH on AXL was then tested by WB upon ZIKV challenge. Vero cells were incubated with Hc-CATH (1.25, 2.5, and 5 μM), AC5 (2.5 μM), LL-37 (2.5 μM), or PBS at 37 °C for 2 h. Vero cells were then washed with PBS and challenged with ZIKV (MOI = 1). After Vero cells were cultured for 6, 12, and 24 h, AXL level was tested by WB analysis.

The effect of Hc-CATH on AXL was further confirmed in A549 and U251 cells. A549 or U251 cells were incubated with Hc-CATH (1.25, 2.5, and 5 μM) or PBS at 37 °C for 24 h, and the level of AXL was detected by WB and IF, respectively. Upon ZIKV challenge, A549 or U251 cells were incubated with Hc-CATH (1.25, 2.5, and 5 μM) or PBS at 37 °C for 2 h. A549 and U251 cells was then washed with PBS and challenged with ZIKV (MOI = 1). After A549 and U251 cells were cultured for 24 h, AXL and NS3 protein level was tested by WB analysis.

### Effect of COX-2/PGE_2_/AC/PKA pathway on Hc-CATH-induced AXL downregulation

A549 cells were cultured with Hc-CATH (1.25, 2.5, and 5 μM) or PBS at 37 °C for 24 h, cells were harvested for detecting the level of COX-2 by WB analysis, and the cell supernatant was collected for detecting the level of PGE_2_ by ELISA.

The effect of Hc-CATH on the enzymatic activity of COX-2 was detected by enzyme activity inhibitor screening kit. Hc-CATH (1.25, 2.5, 5, and 10 μM) or PBS was incubated with COX-2. After incubation at 37 °C for 10 min, the enzymatic activity of COX-2 was detected by the kit. Celecoxib (COX-2 inhibitor, 100 nM) was used as positive control.

To assay the effect of adenylate cyclase (AC) and PKA on Hc-CATH-induced AXL downregulation, we added FSK (agonist of AC, 10 μM) and H89 (inhibitor of PKA, 5 μM) to A549 cells and incubated at 37 °C. After incubation for 1 h, the culture media were removed, cells were washed three times with PBS, and fresh culture media containing 2% FBS were added to cells in the presence of Hc-CATH (5 μM). After incubation for 24 h, AXL protein level was examined by WB.

### Effect of Hc-CATH on the negative regulation of AXL on type I IFN signaling

U251 cells were cultured with Hc-CATH (2.5 μM) or PBS at 37 °C for 24 h, and cells were collected for testing type I IFN signaling gene and protein by qPCR and WB, respectively. Upon ZIKV challenge, U251 cells were incubated with Hc-CATH (2.5 μM) or PBS at 37 °C for 2 h and washed three times with PBS. Cells were then incubated with ZIKV (MOI = 1) for 2 h and washed with PBS. After culture for 24 h, type I IFN signaling genes were tested by qPCR. Upon SeV challenge, U251 cells were incubated with Hc-CATH (2.5 μM) or PBS at 37 °C for 12 h, cells were then stimulated with SeV or same volume of PBS for 12 h, and type I IFN signaling gene and protein were tested by qPCR and WB, respectively.

### Effect of Hc-CATH on ZIKV particles

For assay of the direct inactivation of ZIKV by Hc-CATH, ZIKV (MOI = 1) was incubated with Hc-CATH (2.5 μM), AC5 (2.5 μM), LL-37 (2.5 μM), or same volume PBS at 37 °C for 2 h. ZIKV–PBS mixture and ZIKV–peptide mixture were centrifugated at 100,000*g* for 70 min. The pellets were washed with PBS and centrifugated at 100,000*g* for 70 min again. The pellets were resuspended in PBS and added to Vero cells. The cells were incubated at 37 °C for 2 h, washed with PBS, and transferred with fresh DMEM containing 2% FBS. Then the cells were cultured at 37 °C for 48 h. The intracellular ZIKV RNA, NS3 protein, E protein, and extracellular ZIKV titer were tested by qPCR, WB, IF staining, and plaque-forming assay, respectively (28).

For assay of the binding of Hc-CATH to ZIKV, high-affinity binding plates were coated with 0.25 μM of Hc-CATH, bovine serum albumin, AC5, or LL-37. Then 1 × 10^6^ plaque-forming unit (PFU) of ZIKV was added and incubated. Wells were exposed to anti-ZIKV E protein antibody, horseradish peroxidase–labeled secondary antibody, and TMB substrate in turn. Absorbance at 450 nm was measured after reactions were stopped using 0.5 M sulfuric acid (28).

For assay of the disruption of viral membrane by Hc-CATH, ZIKV (about 10^5^ PFU) was incubated with Hc-CATH (1, 20, and 40 μM), PBS, or 1% Triton X-100 (positive control) at 37 °C for 2 h. The released genomic RNA was digested by micrococcal nuclease at 37 °C for 4 h. The residual RNase was inactivated, the undigested genomic RNA in the intact ZIKV particles was extracted and quantified by testing viral genes encoding membrane protein (PrM), E protein, and capsid (Cap) protein, respectively (43).

### Effect of Hc-CATH on ZIKV replication stage

Vero cells were incubated with ZIKV (MOI = 1) at 37 °C for 2 h, washed three times with PBS, and transferred with fresh DMEM containing 2% FBS and Hc-CATH (2.5 μM), AC5 (2.5 μM), LL-37 (2.5 μM), or PBS. After culture at 37 °C for 48 h, intracellular ZIKV RNA, NS3 protein, E protein, and extracellular ZIKV titer were detected by qPCR, WB, IF, and plaque-forming assay, respectively. The effect of Hc-CATH on ZIKV replication stage was further tested in A549 and U251 cells under the same condition, and intracellular ZIKV NS3 protein was detected by WB.

### Structure–function relationship study

To investigate the role of helical structure of Hc-CATH in its anti-ZIKV activity, the amino acid sequence of Hc-CATH was scrambled to disrupt the α-helix. To investigate the role of aromatic residues of Hc-CATH in its anti-ZIKV activity, phenylalanine residues of Hc-CATH were substituted with alanine residues. To investigate the role of positively charged residues of Hc-CATH in its anti-ZIKV activity, arginine or lysine residues of Hc-CATH were substituted with alanine residues. The anti-ZIKV activity of each mutant peptide was tested and compared with that of Hc-CATH, respectively.

### Antiviral activity of Hc-CATH against ZIKV *in vivo*

To evaluate the prophylactic efficacy of Hc-CATH against ZIKV infection, C57BL/6J mice, *Ifnar1*^−/−^ mice, and pregnant C57BL/6J mice were intravenously administrated with Hc-CATH at 2 h before intravenous injection of ZIKV (10^6^ PFU/mouse). C57BL/6J mice, *Ifnar1*^−/−^ mice, and pregnant C57BL/6J mice were sacrificed at 4, 8, and 3 days post ZIKV inoculation, respectively. Tissues and fetal mice were collected for testing ZIKV RNA level by qPCR.

To evaluate the therapeutic efficacy of Hc-CATH against ZIKV infection, C57BL/6J mice, *Ifnar1*^−/−^ mice, and pregnant C57BL/6J mice were intravenously administrated with Hc-CATH at 2 h after intravenous injection of ZIKV (10^6^ PFU/mouse). C57BL/6J mice, *Ifnar1*^−/−^ mice, and pregnant C57BL/6J mice were sacrificed at 4, 8, and 3 days post ZIKV inoculation, respectively. Tissues and fetal mice were collected for testing ZIKV RNA level by qPCR.

## Statistical analysis

Data were represented as mean ± SD. Statistical significance was determined by an unpaired two-tailed Student’s *t* test for two-group comparison and was determined by ANOVA followed by Bonferroni post hoc analysis for multiple-group comparison. All statistical analyses were performed using GraphPad Prism software, version 5.0 (GraphPad Software, Inc).

## Data availability

All data are contained within the article.

## Supporting information

This article contains supporting information (Tables S1 and S2 and Fig. S1–S6).

## Conflict of interest

The authors declare that they have no conflicts of interest with the contents of this article.

## References

[1] Sirohi D., Kuhn R.J. (2017). Zika virus structure, maturation, and receptors. J. Infect. Dis..

[2] Miner J.J., Diamond M.S. (2017). Zika virus pathogenesis and tissue tropism. Cell Host Microbe..

[3] Gallichotte E.N., Young E.F., Baric T.J., Yount B.L., Metz S.W., Begley M.C. (2019). Role of Zika virus envelope protein domain III as a target of human neutralizing antibodies. mBio.

[4] Lindenbach B.D., Rice C.M. (2003). Molecular biology of flaviviruses. Adv. Virus Res..

[5] Li M.I., Wong P.S., Ng L.C., Tan C.H. (2012). Oral susceptibility of Singapore Aedes (stegomyia) aegypti (linnaeus) to Zika virus. Plos Negl. Trop. Dis..

[6] Deckard D.T., Chung W.M., Brooks J.T., Smith J.C., Woldai S., Hennessey M. (2016). Male-to-Male sexual transmission of Zika virus--texas, january 2016. MMWR Morb Mortal Wkly Rep..

[7] D'Ortenzio E., Matheron S., Yazdanpanah Y., de Lamballerie X., Hubert B., Piorkowski G. (2016). Evidence of sexual transmission of Zika virus. N. Engl. J. Med..

[8] Baud D., Gubler D.J., Schaub B., Lanteri M.C., Musso D. (2017). An update on Zika virus infection. Lancet.

[9] Swaminathan S., Schlaberg R., Lewis J., Hanson K.E., Couturier M.R. (2016). Fatal Zika virus infection with secondary nonsexual transmission. N. Engl. J. Med..

[10] Ma W., Li S., Ma S., Jia L., Zhang F., Zhang Y. (2016). Zika virus causes testis damage and leads to male infertility in mice. Cell.

[11] Hamel R., Dejarnac O., Wichit S., Ekchariyawat P., Neyret A., Luplertlop N. (2015). Biology of Zika virus infection in human skin cells. J. Virol..

[12] Davis C.W., Nguyen H.Y., Hanna S.L., Sanchez M.D., Doms R.W., Pierson T.C. (2006). West Nile virus discriminates between DC-SIGN and DC-SIGNR for cellular attachment and infection. J. Virol..

[13] Tassaneetrithep B., Burgess T.H., Granelli-Piperno A., Trumpfheller C., Finke J., Sun W. (2003). DC-SIGN (CD209) mediates dengue virus infection of human dendritic cells. J. Exp. Med..

[14] Jemielity S., Wang J.J., Chan Y.K., Ahmed A.A., Li W., Monahan S. (2013). TIM-family proteins promote infection of multiple enveloped viruses through virion-associated phosphatidylserine. PLoS Pathog..

[15] Meertens L., Carnec X., Lecoin M.P., Ramdasi R., Guivel-Benhassine F., Lew E. (2012). The TIM and TAM families of phosphatidylserine receptors mediate dengue virus entry. Cell Host Microbe.

[16] Richard A.S., Zhang A., Park S.J., Farzan M., Zong M., Choe H. (2015). Virion-associated phosphatidylethanolamine promotes TIM1-mediated infection by ebola, dengue, and west nile viruses. Proc. Natl. Acad. Sci. U. S. A..

[17] Linger R.M., Keating A.K., Earp H.S., Graham D.K. (2008). TAM receptor tyrosine kinases: Biologic functions, signaling, and potential therapeutic targeting in human cancer. Adv. Cancer Res..

[18] Nowakowski T.J., Pollen A.A., Di Lullo E., Sandoval-Espinosa C., Bershteyn M., Kriegstein A.R. (2016). Expression analysis highlights AXL as a candidate Zika virus entry receptor in neural stem cells. Cell Stem Cell.

[19] Savidis G., McDougall W.M., Meraner P., Perreira J.M., Portmann J.M., Trincucci G. (2016). Identification of Zika virus and dengue virus dependency factors using functional genomics. Cell Rep.

[20] Miner J.J., Diamond M.S. (2016). Understanding how Zika virus enters and infects neural target cells. Cell Stem Cell.

[21] Retallack H., Di Lullo E., Arias C., Knopp K.A., Laurie M.T., Sandoval-Espinosa C. (2016). Zika virus cell tropism in the developing human brain and inhibition by azithromycin. Proc. Natl. Acad. Sci. U. S. A..

[22] Meertens L., Labeau A., Dejarnac O., Cipriani S., Sinigaglia L., Bonnet-Madin L. (2017). Axl mediates ZIKA virus entry in human glial cells and modulates innate immune responses. Cell Rep..

[23] Richard A.S., Shim B.S., Kwon Y.C., Zhang R., Otsuka Y., Schmitt K. (2017). AXL-dependent infection of human fetal endothelial cells distinguishes Zika virus from other pathogenic flaviviruses. Proc. Natl. Acad. Sci. U. S. A..

[24] Lemke G. (2013). Biology of the TAM receptors. Cold Spring Harb Perspect. Biol..

[25] Strange D.P., Jiyarom B., Pourhabibi Zarandi N., Xie X., Baker C., Sadri-Ardekani H. (2019). Axl promotes Zika virus entry and modulates the antiviral state of human Sertoli cells. mBio.

[26] Wei L., Gao J., Zhang S., Wu S., Xie Z., Ling G. (2015). Identification and characterization of the first cathelicidin from sea snakes with potent antimicrobial and anti-inflammatory activity and special mechanism. J. Biol. Chem..

[27] Barlow P.G., Findlay E.G., Currie S.M., Davidson D.J. (2014). Antiviral potential of cathelicidins. Future Microbiol..

[28] Liu Z., Wu J., Qin Z., Dong C., Yang H., Sun J. (2022). Endogenous cathelicidin is required for protection against ZIKV-caused testis damage via inactivating virions. Antivir. Res.

[29] Yu J., Dai Y., Fu Y., Wang K., Yang Y., Li M. (2021). Cathelicidin antimicrobial peptides suppress EV71 infection via regulating antiviral response and inhibiting viral binding. Antivir. Res..

[30] Jenssen H. (2009). Therapeutic approaches using host defence peptides to tackle herpes virus infections. Viruses.

[31] Lee M.T., Sun T.L., Hung W.C., Huang H.W. (2013). Process of inducing pores in membranes by melittin. Proc. Natl. Acad. Sci. U. S. A..

[32] Mulder K.C., Lima L.A., Miranda V.J., Dias S.C., Franco O.L. (2013). Current scenario of peptide-based drugs: The key roles of cationic antitumor and antiviral peptides. Front Microbiol..

[33] Hong W., Lang Y., Li T., Zeng Z., Song Y., Wu Y. (2015). A p7 ion channel-derived peptide inhibits hepatitis C virus infection *in Vitro*. J. Biol. Chem..

[34] Skalickova S., Heger Z., Krejcova L., Pekarik V., Bastl K., Janda J. (2015). Perspective of use of antiviral peptides against influenza virus. Viruses.

[35] Zanetti M. (2004). Cathelicidins, multifunctional peptides of the innate immunity. J. Leukoc. Biol..

[36] Wang Y., Hong J., Liu X., Yang H., Liu R., Wu J. (2008). Snake cathelicidin from Bungarus fasciatus is a potent peptide antibiotics. PLoS One.

[37] Li S.A., Lee W.H., Zhang Y. (2012). Efficacy of OH-CATH30 and its analogs against drug-resistant bacteria *in vitro* and in mouse models. Antimicrob. Agents Chemother..

[38] Pan T., Peng Z., Tan L., Zou F., Zhou N., Liu B. (2018). Nonsteroidal anti-inflammatory drugs potently inhibit the replication of Zika viruses by inducing the degradation of AXL. J. Virol..

[39] Sugimoto Y., Narumiya S. (2007). Prostaglandin E receptors. J. Biol. Chem..

[40] Chen J., Yang Y.F., Yang Y., Zou P., Chen J., He Y. (2018). AXL promotes Zika virus infection in astrocytes by antagonizing type I interferon signalling. Nat. Microbiol..

[41] Coldbeck-Shackley R.C., Eyre N.S., Beard M.R. (2020). The molecular interactions of ZIKV and DENV with the type-I IFN response. Vaccines (Basel).

[42] Wang S., Xie F., Chu F., Zhang Z., Yang B., Dai T. (2017). YAP antagonizes innate antiviral immunity and is targeted for lysosomal degradation through IKKvarepsilon-mediated phosphorylation. Nat. Immunol..

[43] Yu Y., Deng Y.Q., Zou P., Wang Q., Dai Y., Yu F. (2017). A peptide-based viral inactivator inhibits Zika virus infection in pregnant mice and fetuses. Nat. Commun..

[44] Duan Z., Zhang J., Chen X., Liu M., Zhao H., Jin L. (2022). Role of LL-37 in thrombotic complications in patients with COVID-19. Cell Mol Life Sci.

[45] Holthausen D.J., Lee S.H., Kumar V.T., Bouvier N.M., Krammer F., Ellebedy A.H. (2017). An Amphibian host defense peptide is virucidal for human H1 hemagglutinin-bearing influenza viruses. Immunity.

[46] He M., Zhang H., Li Y., Wang G., Tang B., Zhao J. (2018). Cathelicidin-derived antimicrobial peptides inhibit Zika virus through direct inactivation and interferon pathway. Front. Immunol..

[47] Li S., Zhu A., Ren K., Li S., Chen L. (2020). DEFA1B inhibits ZIKV replication and retards cell cycle progression through interaction with ORC1. Life Sci..

[48] Ji Z., Li F., Xia Z., Guo X., Gao M., Sun F. (2018). The scorpion venom peptide Smp76 inhibits viral infection by regulating type-I interferon response. Virol. Sin.

[49] Li F., Lang Y., Ji Z., Xia Z., Han Y., Cheng Y. (2019). A scorpion venom peptide Ev37 restricts viral late entry by alkalizing acidic organelles. J. Biol. Chem..

[50] Ji M., Zhu T., Xing M., Luan N., Mwangi J., Yan X. (2019). An antiviral peptide from Alopecosa nagpag spider targets NS2B-NS3 protease of flaviviruses. Toxins (Basel).

[51] Lee S.H., Kim E.H., O’Neal J T., Dale G., Holthausen D.J., Bowen J.R. (2021). The amphibian peptide Yodha is virucidal for Zika and dengue viruses. Sci. Rep..

[52] Gonzalez Garcia M., Rodriguez A., Alba A., Vazquez A.A., Morales Vicente F.E., Perez-Erviti J. (2020). New antibacterial peptides from the freshwater mollusk Pomacea poeyana (pilsbry, 1927). Biomolecules.

[53] Mookherjee N., Anderson M.A., Haagsman H.P., Davidson D.J. (2020). Antimicrobial host defence peptides: Functions and clinical potential. Nat. Rev. Drug Discov..

[54] Xing M., Ji M., Hu J., Zhu T., Chen Y., Bai X. (2020). Snake cathelicidin derived peptide inhibits Zika virus infection. Front Microbiol..

[55] Yang Y., Liu Z., He X., Yang J., Wu J., Yang H. (2019). A small mycobacteriophage-derived peptide and its improved isomer restrict mycobacterial infection via dual mycobactericidal-immunoregulatory activities. J. Biol. Chem..

[56] McNab F., Mayer-Barber K., Sher A., Wack A., O'Garra A. (2015). Type I interferons in infectious disease. Nat. Rev. Immunol..

