# Supplementary material for: A cathelicidin antimicrobial peptide from Hydrophis cyanocinctus inhibits Zika virus infection by downregulating expression of a viral entry factor
Source: J Biol Chem. 2022 Sep 8;298(10):102471. doi: 10.1016/j.jbc.2022.102471 (PMC9530963; doi:10.1016/j.jbc.2022.102471)
Supplement: Supporting Information [file mmc1.pdf]

Supplementary tables

Table S1. Amino acid sequence of Hc-CATH and the positive control peptides

Peptide	Amino acid sequence
Hc-CATH	KFFKRLLKSVRRAVKKFRKKPRLIGLSTLL
AC5	GGLKKLGKKLEGAGKRVFKASEKALPVVVGIKAI-NH_2
LL-37	LLGDFFRKSKEKIGKEF-KRIVQRIKDFLRNLVPRTES

Table S2. Primers for qPCR.

**Primer**	**Sequence (5'→3')**
mouse β-actin forward	AACAGTCCGCCTAGAAGCAC
mouse β-actin reverse	CGTTGACATCCGTA-AAGACC
ZIKV forward	GCTGCCCAACACAAGGTGAA
ZIKV reverse	ATGTCACCAGGCTCCCTTTG
African green monkey β-actin forward	GCCGGGACCTGACTGACTAC
African green monkey β-actin reverse	ATCGGGCAGCTCGTAGCTCT
PrM forward	CTTGGA CAGAAACGATGCTGGG
PrM reverse	TGATGGCAGGTTCCGTACACAA
E forward	TGGAGGCTGAGATGGATGG
E reverse	GAACGCTGCGG TACACAAGGA
Cap forward	TCACGGCAATCAAGCCATCACT
Cap reverse	GCCTCGTCTCTTCTTCTCCTT
human β-actin forward	TGGAGAAAATCTGGCACCACACC
human β-actin reverse	GATGGGCACAGTGTGGGTGACCC
human IFNβ forward	GAGCTACAACTTGCTTGGATTC
human IFNβ reverse	CAAGCCTCCCATTCAATTGC
human IFNω1 forward	GAAGGCCCATGTCATGTCTGT
human IFNω1 reverse	GAGTTGGTCTAGGAGGGTCAT
human IFNκ forward	GTGGCTTGAGATCCTTATGGGT
human IFNκ reverse	CAGATTTTGCCAGGTGACTCTT
human IFNε forward	GGCCTCTACCACTATCTTCTCTC
human IFNε reverse	ACACTGCTGAATTGACAAGGTTT
human IFNα1 forward	GCCTCGCCCTTTGCTTTACT
human IFNα1 reverse	CTGTGGGTCTCAGGGAGATCA
human IFNα2 forward	GCTTGGGATGAGACCCTCCTA
human IFNα2 reverse	CCCACCCCCTGTATCACAC
human IFNα2b forward	GCTTGGGATGAGACCCTCCTA
human IFNα2b reverse	CCCACCCCCTGTATCACAC
human IFNα7 forward	AGGGCCTTGATACTCCTGG
human IFNα7 reverse	TCCTCCTCCGGGAATCTGAAT
human IFNα21 forward	AGGGCCTTGATACTCCTGG
human IFNα21 reverse	TCTTGGGGGAATCCAAAGTCA

Supplementary figures and figure legends

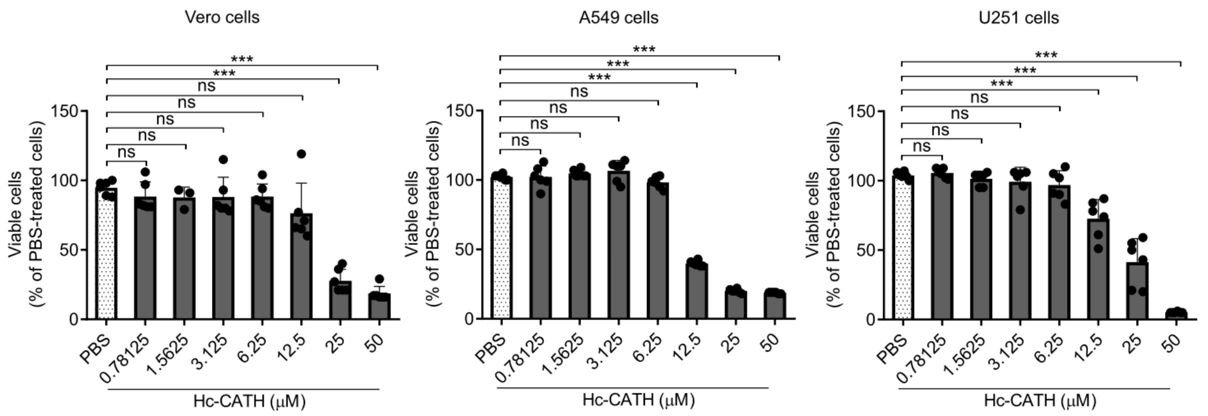

Fig. S1. **Cytotoxicity of Hc-CATH.** Vero, A549 or U251 cells (1×10^4 cells/well) were seeded in 96-well plates. Series of two-fold peptide dilutions in DMEM were added to each well to give final concentrations from 0.78125 to 50 μM. After incubation at 37℃ for 48 h, cell counting assay kit (CCK-8) was applied to determine the cytotoxicity. ***$P < 0.001$, ns, not significant.

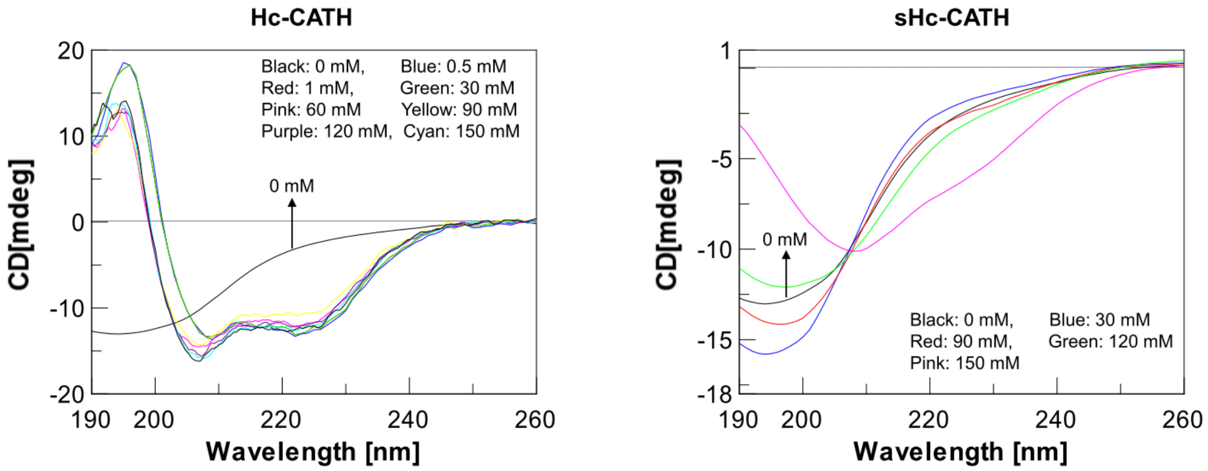

Fig. S2. **Circular dichroism experiment was performed to demonstrate that the scrambled Hc-CATH has effectively lost its helicity.** Hc-CATH and scrambled Hc-CATH (0.2 mg/mL) was prepared in H_2O (0 mM SDS) and SDS/H_2O as indicated. CD spectra were recorded at 298 K on a Jasco-810 spectropolarimeter (Jasco, Tokyo, Japan) with 1 mm path-length cell and 0.2 nm interval from 190 to 260 nm. Data from three consecutive scans were averaged, smoothed and expressed as the mean residue ellipticity (θ) in deg·cm^2 ·dmol^{-1}.

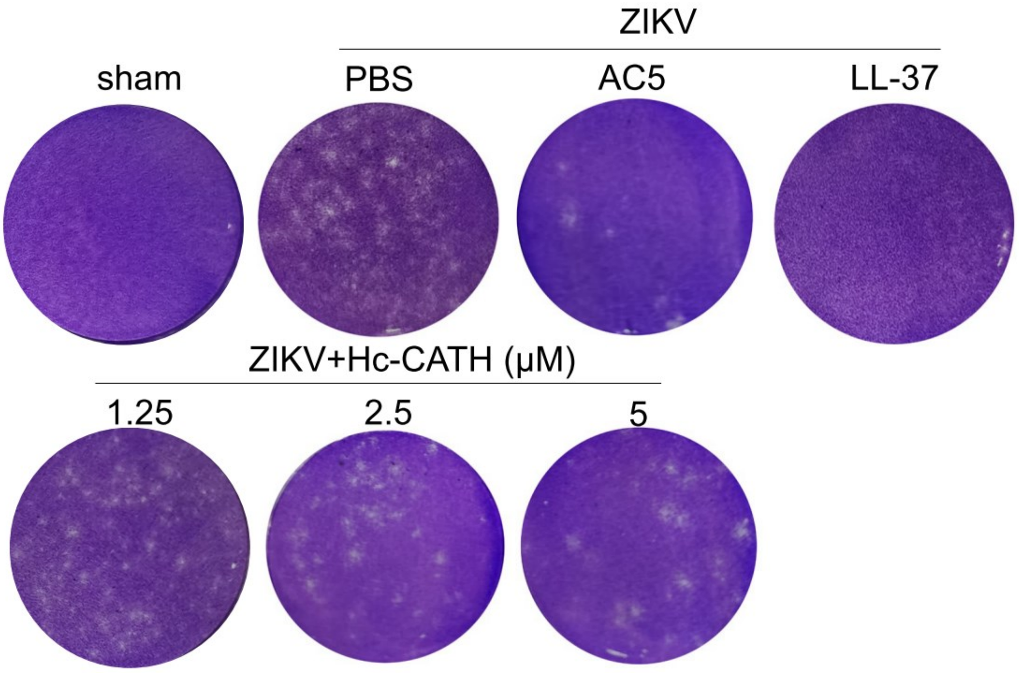

Fig. S3. **The raw data files used in the creation of figure 1G.**

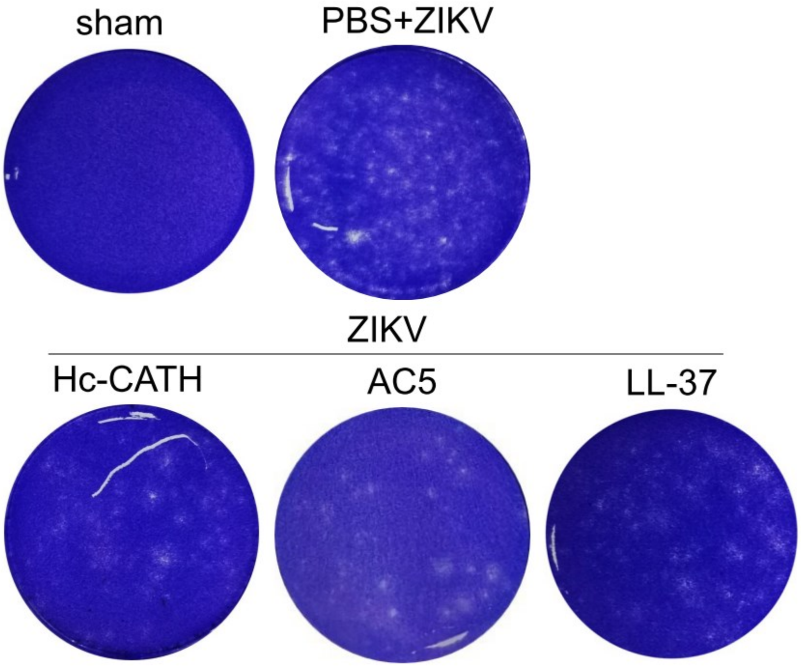

Fig. S4. **The raw data files used in the creation of figure 2F.**

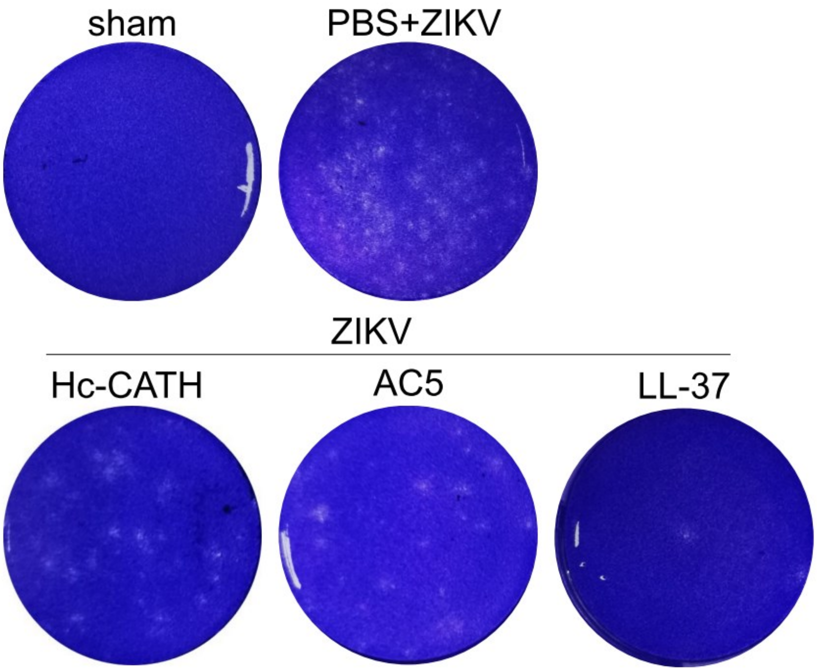

Fig. S5. **The raw data files used in the creation of figure 8F.**

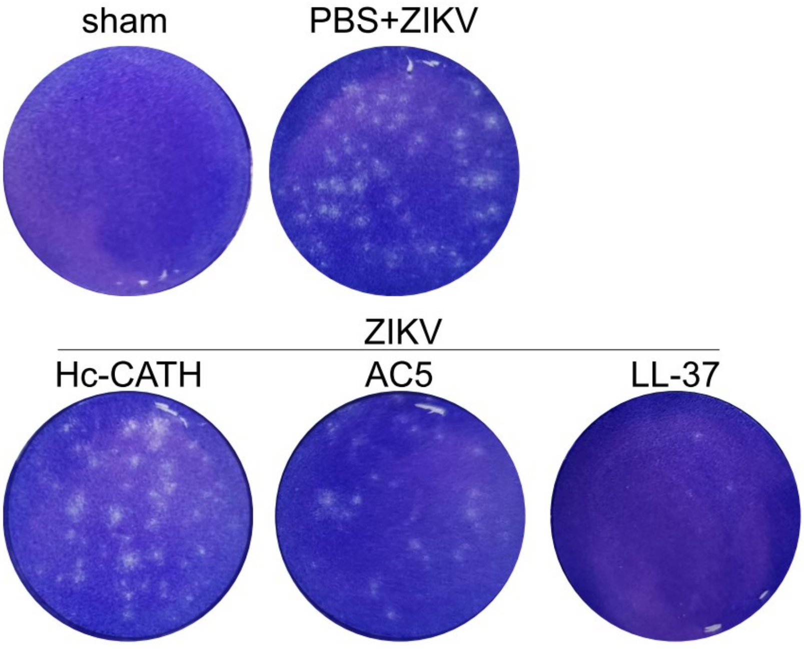

Fig. S6. **The raw data files used in the creation of figure 9F.**
